# Modeling membrane geometries implicitly in Rosetta

**DOI:** 10.1002/pro.4908

**Published:** 2024-02-15

**Authors:** Hope Woods, Julia Koehler Leman, Jens Meiler

**Affiliations:** ^1^ Center of Structural Biology, Vanderbilt University Nashville Tennessee USA; ^2^ Chemical and Physical Biology Program Vanderbilt University Nashville Tennessee USA; ^3^ Center for Computational Biology Flatiron Institute New York New York USA; ^4^ Department of Chemistry Vanderbilt University Nashville Tennessee USA; ^5^ Institute for Drug Discovery, Leipzig University Medical School Leipzig Germany

**Keywords:** implicit membrane, membrane proteins, protein structure, Rosetta

## Abstract

Interactions between membrane proteins (MPs) and lipid bilayers are critical for many cellular functions. In the Rosetta molecular modeling suite, the implicit membrane energy function is based on a “slab” model, which represent the membrane as a flat bilayer. However, in nature membranes often have a curvature that is important for function and/or stability. Even more prevalent, in structural biology research MPs are reconstituted in model membrane systems such as micelles, bicelles, nanodiscs, or liposomes. Thus, we have modified the existing membrane energy potentials within the RosettaMP framework to allow users to model MPs in different membrane geometries. We show that these modifications can be utilized in core applications within Rosetta such as structure refinement, protein–protein docking, and protein design. For MP structures found in curved membranes, refining these structures in curved, implicit membranes produces higher quality models with structures closer to experimentally determined structures. For MP systems embedded in multiple membranes, representing both membranes results in more favorable scores compared to only representing one of the membranes. Modeling MPs in geometries mimicking the membrane model system used in structure determination can improve model quality and model discrimination.

## 1 INTRODUCTION

Membrane proteins (MPs) exist in complex and diverse membrane environments in which they function. Cellular membranes adopt different geometries and cover a variety of different lipid compositions which affect membrane thickness. Lipid composition, thickness and curvature vary depending on species, cell type, and organelle the membrane belongs to (Findlay & Booth, 2006; Harris et al., 2018; van Meer et al., 2008). Protrusions and invaginations of the membrane, such as filopodia or caveolae, result from local membrane curvature in a larger bilayer. Vesicles, important for transport of various cargo molecules, are another classic example of curved membranes. Further, some MPs are large complexes that insert into two membranes in close proximity, such as gap junction channels across two cells (Myers et al., 2018) or efflux pumps that are multi‐protein complexes that traverse the periplasm and insert into both the outer and inner membrane in Gram‐negative bacteria (Wang et al., 2017).

Interactions between MPs and their lipid bilayer affect one another's shape, stability, and function (Findlay & Booth, 2006; Kleinschmidt & Tamm, 2002; van den Brink‐van der Laan et al., 2004). Some MPs modify the membrane they are in, either by changing their thickness (e.g., some GPCRs; Gahbauer & Bockmann, 2016), curvature (e.g., BAR domains; Hansen et al., 2011), lipid composition (e.g., flippases, floppases, and scramblases; Daleke, 2007) or recruiting specific lipids to their location (e.g., some channels such as aquaporin Z (AqpZ) and the ammonia channel (AmtB); Laganowsky et al., 2014). Reversely, the membrane bilayer can directly affect the function of many MPs such as in ABC transporters, RTKs, and mechanosensitive channels including some channels from the Piezo, TRP, MscL, and TREK families (Corin & Bowie, 2020; Dong et al., 2010; Harayama & Riezman, 2018; Harris et al., 2018; Iscla & Blount, 2012; Jarsch et al., 2016; Noel et al., 2011; Zhou et al., 2019).

Because structure determination of MPs in native membranes is practically challenging, MPs are typically solubilized into model membrane systems that allow for structure determination. These systems involve artificial membrane geometries that are rarely or never seen in cellular environments. Commonly used model membrane systems include detergent micelles, mixed detergent and lipid bicelles, lipid nanodiscs, and liposomes which vary in molecular composition (Choy et al., 2021; Zhou & Cross, 2013). The choice of model membrane systems can impact the structure and stability of the MP (Kim et al., 2009; Poget et al., 2007; Zhou & Cross, 2013). Unfortunately, the model membrane system geometry in which experiments are performed is often disregarded during structure determination and is instead replaced by a flat bilayer (see below).

Despite significant progress in recent years, MP structure prediction and design lags behind that of soluble proteins (Barth et al., 2007). Recent machine learning algorithms for protein structure prediction and design, such as AlphaFold2 (Jumper et al., 2021a; Jumper et al., 2021b) and ProteinMPNN (Dauparas et al., 2022), neglect the membrane environment, which may cause inaccurate predictions in some cases (Dobson et al., 2023). Because state‐of‐the‐art machine learning techniques depend on large amounts of training data, the vast under‐representation of MP structures in the Protein Data Bank (PDB) as compared to soluble proteins (Kozma et al., 2013; Tusnady et al., 2004) (2.7% vs. 97.8% or 36‐fold) is most likely the main reason for the limitations of these methods when it comes to MPs.

Computational modeling of membrane bilayers is achieved using explicit or implicit solvent. Molecular dynamics simulations often use explicit solvent models where diverse lipid bilayers or model membrane systems are modeled by representing individual lipid or detergent molecules, which is computationally expensive (Ulmschneider & Ulmschneider, 2009). In contrast, implicit membrane energy functions, like those used in Monte‐Carlo techniques such as the Rosetta software, are computationally efficient and model the effects the membrane has on protein structures (Alford et al., 2020; Barth et al., 2007; Lazaridis, 2003) through the interaction of the protein with a continuous medium of average bilayer properties. Implicit membrane models such as IMM1 (Lazaridis, 2003) are useful for many applications, including MP structure prediction, protein–protein docking, and design (Alford et al., 2015; Alford et al., 2020; Duran & Meiler, 2018; Leman et al., 2015). While much progress has been made in improving these models to represent the membrane environment more accurately, most implicit membrane models are limited to a flat representation of the membrane, or a “slab” model. The slab neglects the fact that membrane proteins may exist and function in different geometries such as highly curved membranes with a radius of curvature as low as 50 Å (Jarsch et al., 2016), model membrane systems such as micelles, bicelles, nanodiscs, or double membranes.

Recent improvements to the Rosetta implicit membrane allow more realistic bilayer representations by customizing parameters to model different lipid compositions (Alford et al., 2020). This update included the ability to model aqueous pores in MPs. One limitation of this membrane model is the lack of geometrical diversity it can simulate. One method and web server, PPM 3.0 (“Positioning of Proteins in Membranes”), accounts for different membrane geometries such as curvature or two membranes (Lomize et al., 2022). PPM 3.0 evaluates whether a protein is more likely to exist in a flat or curved membrane based on the transfer free energy from water to the membrane, calculated from the protein structure. The PPM server runs a grid search of a MP placed in a flat membrane and a curved membrane testing different radii of curvature from 80 to 600 Å, to find the minimum transfer free energy and therefore the ideal membrane geometry for the protein structure in question. In case the MP is embedded in two or more membranes, it can give the optimal relative placement of each bilayer around the protein. The authors provide many examples of MP structures that were optimally placed in curved membranes or in multiple membranes (Lomize et al., 2022). One can input their protein structure of interest into the PPM webserver or look up the PDB ID in the OPM (“Orientations of Proteins in Membranes”) database to see the predicted optimal membrane placement (Lomize et al., 2006; Lomize et al., 2012). FMAP (“Folding of Membrane Associated Peptides”), uses the peptide sequence to predict helical propensity, membrane depth, and ideal membrane geometry for different environments, water, lipid bilayer, or a micelle with a fixed radius depending on detergent (Lomize et al., 2021). Both PPM 3.0 and FMAP are limited to finding the optimal membrane geometrical parameters for placing the protein into the membrane. Further modeling such as structure refinement, docking or protein design, will need to be executed with different software tools such as Rosetta or molecular dynamics packages.

Here we introduce a substantial code refactor within the RosettaMP framework to allow the use of different geometries in the implicit membrane model. These geometries include the traditional flat model, an ellipsoid model to represent model membrane system geometries such as micelles, bicelles, and nanodiscs, a spherical bilayer like a vesicle to simulate curvature, and a model with two membranes. We provide examples of how using these models improve computational predictions for protein design, protein–protein docking, and high‐resolution refinement, showcasing how these models can be applied to a variety of protocols within Rosetta. These are common tasks performed with Rosetta that can be incorporated into more complex protocols based on specific use cases.

## 2 RESULTS

### 2.1 We added different implicit membrane geometries to RosettaMP

This substantial refactor was geared to achieve multiple goals: (1) adapting the current implicit membrane models for geometries that mimic micelles, bicelles, vesicles, membrane curvature, and double bilayers, (2) adapting the code interface to facilitate implementation of new geometries (e.g., lipidic cubic phases), (3) streamlining the current implementation by disentangling the pore from membrane geometries to be used independently and creating a membrane representation from a single, consistent implementation, thereby (4) minimizing code duplication, (5) improving testing, and (6) visualizing membrane geometries onto protein structures.

### 2.2 Previous implicit membranes in Rosetta only describe a flat membrane

**FIGURE 1:**
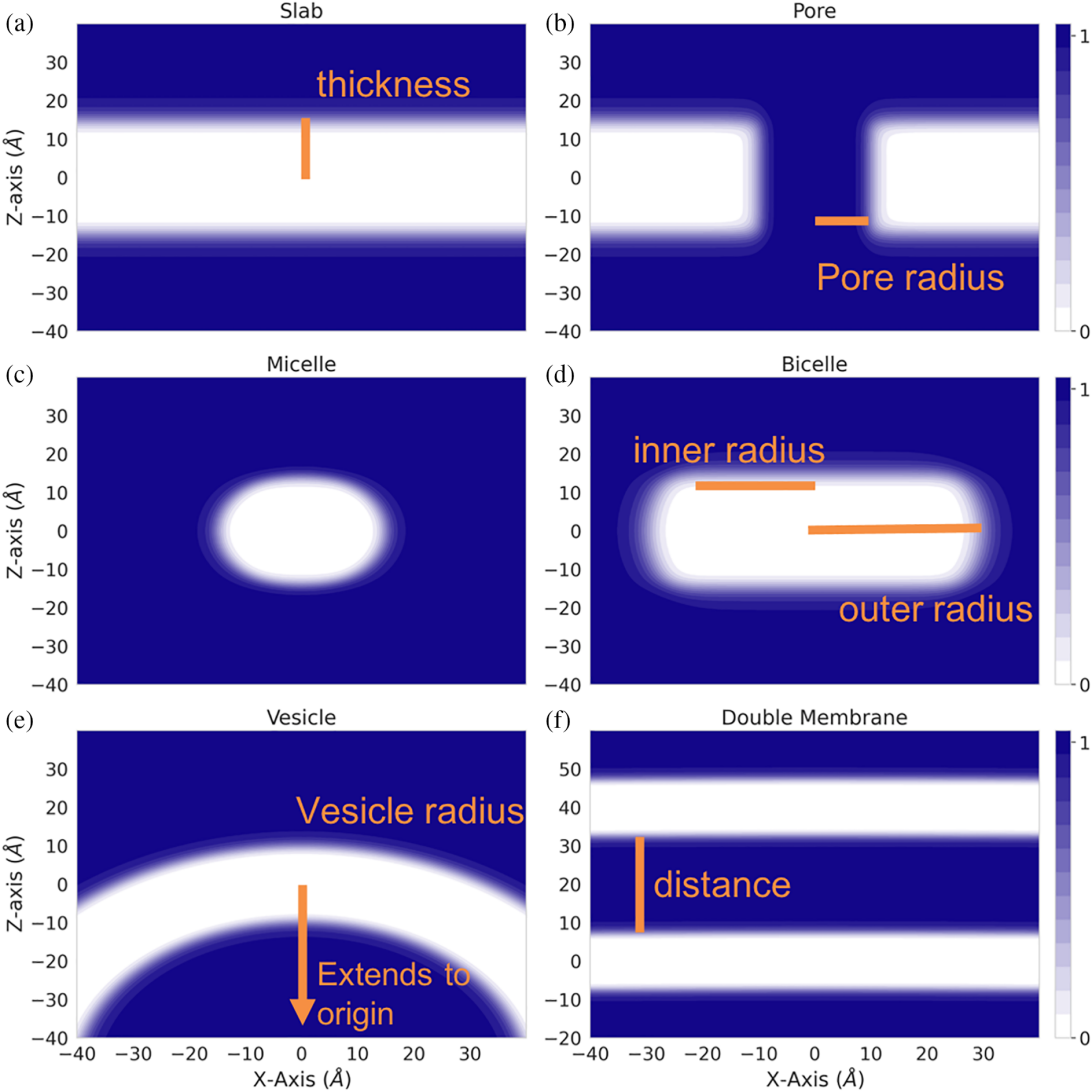
Implicit membrane energy transition functions. Value of transition functions for different geometries are shown in the *XZ* plane, at *Y* = 0. Atoms with transition function values of 0 (white) are evaluated as being in a completely hydrophobic environment. Atoms with transition functions values of 1 (blue) are evaluated as being in a hydrophilic environment. Atoms with transition function values in between are weighted based on their depth in the membrane. Input parameters are shown in orange. (a) Slab and (b) slab including a pore. (c) Micelle, inner radius = 0, (d) bicelle, (e) vesicle, and (f) double membrane.

Mathematically, the membrane is modeled by a transition function that describes the transition from the aqueous environment to the hydrophobic environment of the membrane. Two transition functions had previously been implemented, one based on IMM1 and an adaptation to model different lipid compositions. Disregarding the pore, both transition functions score atoms based on their distance from the center of a membrane plane, meaning that the hydrophobicity at a specific position is only dependent on the depth in the membrane (*z*‐coordinate). This creates the implicit slab membrane (Figure 1a).

### 2.3 Expanding beyond flat membranes through implementation of mathematical representations of desired geometries

To model different geometries, transition functions must depend on the distance to the membrane center in more than one dimension. The pore was the first adaptation that introduced a three‐dimensional dependence (Figure 1b). In order to create an ellipsoid geometry similar to micelles and bicelles, we created a functional form that combines the slab transition function (He et al., 2013) with a function that depends on the distance to the center of the ellipsoid (Figure 1c,d). Although micelles and bicelles have different chemical and physical properties, we use the same function to describe their shape whereas the micelle is a special case of the bicelle with an inner radius of the ellipsoid (Figure 1d) just large enough to surround the transmembrane region of the protein. To model a curved membrane such as a vesicle, we model the membrane as a sphere with the sphere's center at (0, 0, −R), with R being the radius of the sphere or vesicle. The protein is embedded in the membrane around the origin (0, 0, 0) which is at the center of the membrane. Therefore, location of the membrane residue used in the RosettaMP framework, which stores information about the implicit membrane position and orientation, remains the same with a structure already transformed into membrane coordinates using the RosettaMP framework (Alford et al., 2015). The vesicle concept is extended to describe two membranes by a double vesicle (two concentric spheres) that, with a large radius, represent two essentially flat membranes (Figure 1f). Detailed descriptions of equations and parameters can be found in Section 5.

### 2.4 Code design ensures current and new geometries are compatible across membrane energy functions

To achieve our goal of generalizability across score functions and simplify the implementation of new geometries, we introduced an abstract base class into the RosettaMP framework that all other membrane geometry classes are derived from (Figure S1). By defining pure virtual functions within the MembraneGeometry base class, we ensure that all derived classes (or new geometries) define the transition function and the derivative of the transition function for that geometry. Functions used across different geometry classes are defined in the MembraneGeometry base class to decrease code duplication. For example, the functions for describing the pore are now defined in the MembraneGeometry class where they can be combined with different transition functions and geometries while keeping the equations describing the pore environment the same as originally implemented (Alford et al., 2020). Implementation details, function definitions, and score terms that depend on them, can be found in Section 5. This implementation ensures that new geometries can be implemented by solely creating a class and updating options for setting the new geometry without updating each individual score term that depends on the transition function. This also allows for geometries to be used across score terms without the need to define the geometries or transition functions separately for each score term, including the equations describing the pore. Therefore, membrane‐dependent score terms that use a transition function implemented before the pore can utilize the pore model as well.

### 2.5 Optimized code design allows for integrating membrane geometries into different applications

Implementation of the membrane geometries following the same object oriented design principles used in the RosettaMP framework allows for use in any application accessible to the RosettaMP framework (Alford et al., 2015), including but not limited to design, refinement, and protein–protein docking. This implementation also ensures a consistent user interface with the same command line options for setting geometry parameters across applications. The membrane geometries are also accessible through the different Rosetta scripting interfaces, including RosettaScripts (Fleishman et al., 2011) and PyRosetta (Chaudhury et al., 2010).

### 2.6 New implementation decouples pore model from energy functions, as tested for design

**FIGURE 2:**
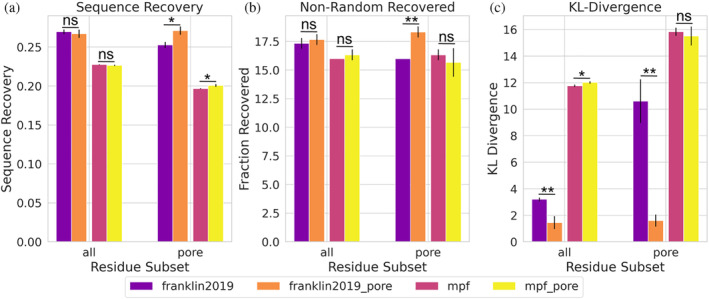
Sequence design of pore across score functions. Our implementation allows the decoupling of the pore model from the energy functions, here shown for franklin2019 and mpframework2012 (mpf). This data shows that the improvements for design originate in the franklin2019 energy function where the pore model only plays a minor role in the improvements. (a) Fraction of native residues recovered after design for all residues (left) and pore‐facing residues (right). Higher values are expected for more accurate energy functions. Results using the franklin2019 function are purple, franklin2019 with the pore are orange, mpframework2012 are pink, mpframework2012 are yellow. (b) Average recovery rates for each individual amino acids relative to the background probability of randomly guessing the native amino acid (1/20). Higher values are expected for more accurate energy functions. (c) Kullback–Leibler (KL) divergence measures how different distributions are of designed amino acids compared to native distributions. Lower values are expected for more accurate energy functions.

Alford et al. described an improvement in protein design using their newly developed energy function, *franklin2019*, compared to previous membrane energy functions (Alford et al., 2020). However, it was not clear if these improvements came from the new energy function, from the inclusion of the pore, or a combination of the two. This could not be tested because the pore was incompatible with older membrane energy functions. Our reimplementation of the pore function into the MembraneGeometry class allows for its use across applications and energy functions. Therefore, we are able to test protein design including the pore for both energy functions, *mpframework2012* and *franklin2019*. We used an established membrane protein design benchmark set to test where this improvement comes from (Alford et al., 2021). The test uses three different metrics to describe performance: (Alford et al., 2020) sequence recovery is the fraction of native residues recovered after design over all designable positions. (Alford et al., 2015) non‐random recovery of individual amino acids, where the recovery rate for each amino acid is calculated relative to the background probability of randomly guessing the native amino acid (1/20). (Alford et al., 2021) The Kullback–Leibler (KL) divergence measures how different distributions of designed amino acids are compared to native distributions. The design test done on the subset of proteins from the dataset that contain a pore to highlight the effect of accounting for an aqueous pore. The design protocol was run with six different energy function conditions: *mpframework2012* without the pore included, *mpframework2012* with the pore, *franklin2019* without the pore, *franklin2019* with the pore, *ref2015_memb (*Weinstein et al., 2019 *)*, and *ref2015* (Figures 2 and S2). When analyzing all residues in each protein, *franklin2019* outperforms the *mpframework2012* energy function with respect to all three metrics. Inclusion of the pore in either *franklin2019* or *mpframework2012* score functions does not improve the sequence recovery or non‐random recovery when looking at all residues, but improvement in the KL‐divergence is observed for *franklin2019* (Figure 2). The *franklin2019* with the pore outperforms *mpframework2012* based on all three metrics for all residue subsets tested, except non‐random recovery for lipid facing residues where there is no significant difference (Figure S2). If only pore‐facing residues are taken into account, inclusion of the pore for *franklin2019* shows a significant difference in all three metrics. Differences in performance when including the pore for *mpframwork2012* are minor. An improvement in KL divergence is seen with *franklin2019* across all residues, and the difference is greater when only considering pore‐facing residues (Figure 2c). On the other hand, KL divergence does not show an improvement for *mpframework2012* when including the pore.

### 2.7 Refinement of structures in a curved membrane results in higher quality models

**FIGURE 3:**
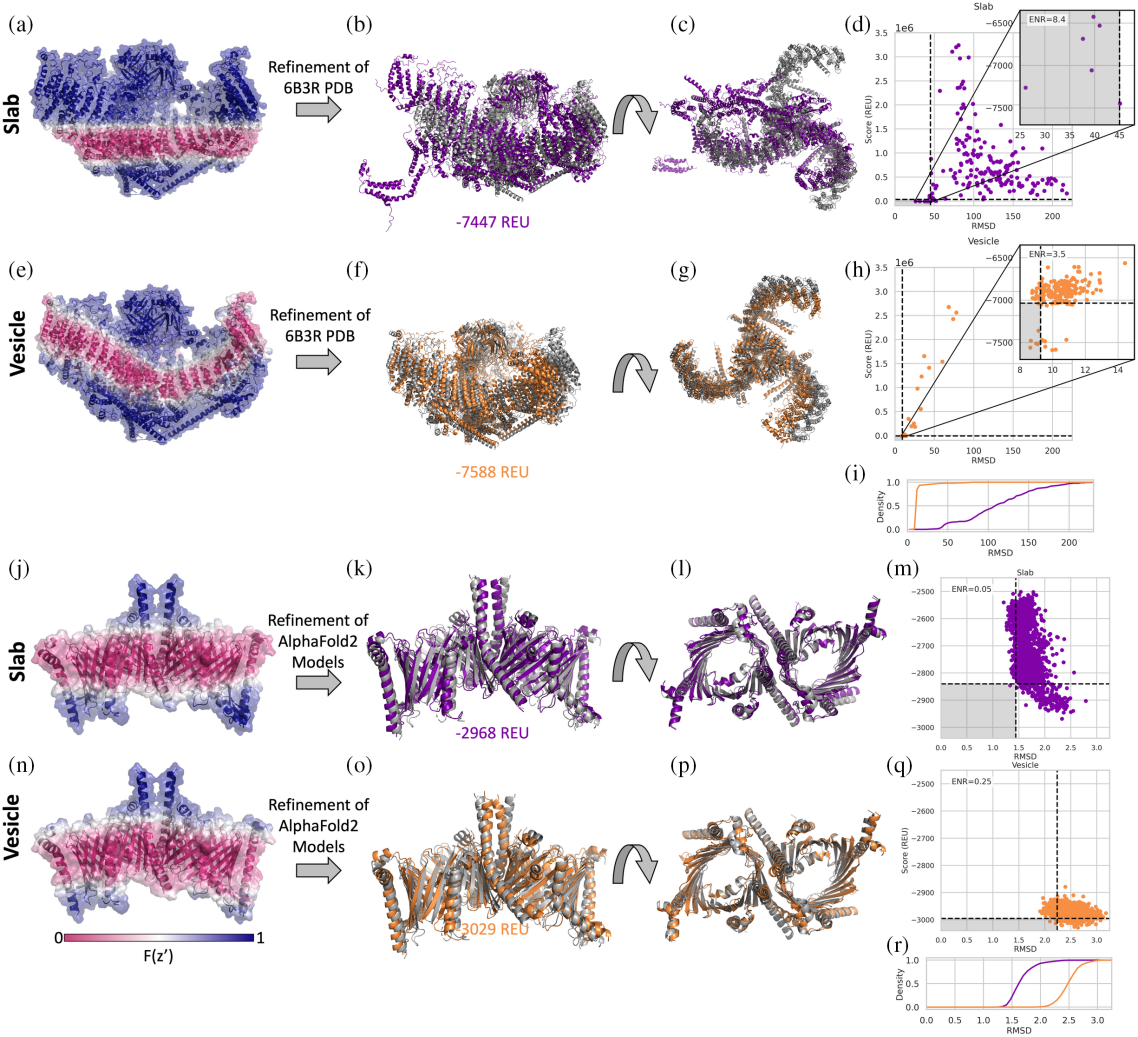
Refinement in vesicle geometry. (a) Mechanosensitive channel Piezo 1 (PDB ID 6B3R) with the slab transition function mapped onto the structure. A value of zero is shown in pink and indicates that residue is scored in a hydrophobic environment. A value of one is show in blue and indicates that residue is scored in an aqueous environment. (b) Lowest scoring structure after refinement with slab geometry ran on PDB structure with a score of −7447 Rosetta Energy Units (REU). Starting structure (PDB ID 6B3R) shown in gray. (c) Top view of structure in (b). (d) C‐alpha RMSD with respect to PDB 6B3R vs. score of models from refinement with slab geometry, inset plot shows lowest scoring models, with enrichment (ENR) value of 8.4. Below the horizontal black dotted line are models that score in the lowest 10% and to the left of the vertical black dotted line are models with the lowest 10% RMSD values. (e) Same structure as (a) with vesicle geometry transition function with a radius of 120 Å. (f) Lowest scoring structure after refinement with vesicle geometry ran on PDB structure with a score of −7588 REU. (g) Top view of structure in (f). (h) RMSD with respect to PDB 6B3R vs. score of models from refinement with vesicle geometry, inset plot shows lowest scoring models, with ENR value of 3.5. (i) Cumulative distribution of RMSD of decoys produced by slab (purple) and vesicle (orange) geometries Piezo 1. (j) Mitochondrial TOM complex (PDB 6UCU) with the slab transition function mapped onto the structure. (k) Lowest scoring structure from generating structures with AlphaFold2 followed by refinement in Rosetta with slab geometry ran on PDB structure with a score of −2968 (REU). Starting structure (PDB ID 6UCU) shown in gray. (l) Top view of structure in (j). (m) RMSD with respect to PDB 6UCU vs. score of models from AlphaFold2 followed by refinement with slab geometry with ENR value of 0.05. (n) Same structure as (i) with vesicle geometry transition function with a radius of 180 Å. (o) Lowest scoring structure from generating structures with AlphaFold2 followed by refinement in Rosetta with slab geometry ran on PDB structure with a score of −3029 (REU). (p) Top view of structure in (n). (q) RMSD with respect to PDB 6UCU vs. score of models from AlphaFold2 followed by refinement with vesicle geometry, with an ENR value of 0.25. (r) Cumulative distribution of RMSD of decoys produced by slab (purple) and vesicle (orange) geometries of TOM complex.

Several MPs exist in curved membranes. To take curvature into account we have implemented a vesicle geometry where the user would set the vesicle radius to define the degree of curvature. The vesicle radius is the distance from the center of the vesicle to the center of the membrane. We tested refinement in a curved membrane on the mechanosensitive channel Piezo 1 (PDB ID 6B3R). The curve of the detergent micelle around Piezo 1 can clearly be seen in the cryo‐EM images (fig. 2 in Guo & MacKinnon, 2017). When the slab transition function is mapped onto the structure it is clear that it does not properly represent the hydrophobic environment needed (Figure 3a). The result of running high‐resolution refinement with this poor membrane placement is evident from the large structural changes in the output models as compared to the input structure (Figure 3b–d), refinement essentially breaks up the protein. Even the lowest scoring model with the slab geometry has an RMSD of 26 Å with large changes compared to the starting structure shown in gray (Figure 3b–d). The vesicle transition function with a radius of 120 Å (based on PPM 3.0 predictions) better fits the membrane geometry in Piezo 1 (Figure 3e). The lowest scoring model is much closer to the starting structure for the vesicle geometry (~8Å, Figure 3f,g). While both geometries also produce models that have large positive scores due to clashes, the percentage of low‐scoring, high‐quality models is much higher for the vesicle geometry than for the slab (Figure 3d,h). Enrichment is a scoring metric measuring how many of the 10% most favorable models based on score (horizontal dotted line in Figure 3d,h) also are part of the top 10% based on RMSD (vertical dotted line in Figure 3d,h); if the top 10% of both categories overlap completely the enrichment will be 10 and if none of them are the same the enrichment is 0 (Kuenze et al., 2019). In this case, the slab model has a higher enrichment value indicating the change in scoring may not be improving the discrimination power. However, quality of models sampled is highly improved (Figure 3i) and the higher enrichment value for the slab model may be an artifact of their being so few low RMSD models similar. In another curved membrane example of potassium chloride cotransporter KCC2, the slab geometry also results in more high scoring, low‐quality models (Figure S3c,f). In this case though, the slab geometry still also produces several low RMSD, low scoring models, potentially because the difference in the implicit membrane from the slab and vesicle geometries are not as great as for Piezo 1 (Figure S3a,d). ThFe enrichment values are similar in this case, but there are more lower RMSD models sampled (Figure S3g).

### 2.8 Refinement and scoring of AlphaFold2 models in a curved membrane

The TOM complex (translocase of the outer membrane) was recently determined by cryo‐EM (PDB ID 6UCU) (Tucker & Park, 2019). PPM 3.0 predicts that TOM complexes induce membrane curvature with a radius of ~180 Å (Lomize et al., 2022). How the TOM complex structure would be scored in the slab and vesicle geometries can be seen by mapping the transition function values onto the structure (Figure 3j,n). As a demonstration of how one could use this implementation in combination with other tools, we used AlphaFold2 multimer (Richard et al., 2022) to predict the structure of the TOM complex from the sequence. Then, we used the FastRelax protocol to optimize and score the predicted models in the slab and the vesicle implicit membrane geometries. The lowest scoring models for both slab (Figure 3j,k) and vesicle geometries (Figure 3o,p) are similar to the determined structure shown in gray. While the slab geometry results in output models with slightly lower RMSD values to the PDB structure (Figure 3r), they have a much larger range of scores, indicating that the refinement cannot find an ideal fit of the flat membrane around the protein. Almost all the models from the vesicle geometry have lower scores than the models from the slab geometry (Figure 3l,p), indicating a better fit of the protein in the vesicle than in the slab model.

### 2.9 Modeling two membranes provides a more accurate representation of membrane protein systems spanning different bilayers

Another limitation of previous implicit membrane energy functions is only being able to account for one membrane per system. While one membrane is often sufficient, this ignores cases where one protein complex spans two membranes, such as in gram negative bacteria. Here we introduce a double vesicle option that represents the membrane as two vesicles, sharing a single origin but with different radii. One can approximate two flat membranes by setting a large radius for the vesicles, such as 1000 Å.

### 2.10 Implicit membrane for lipid bilayers of two interacting cells

**FIGURE 4:**
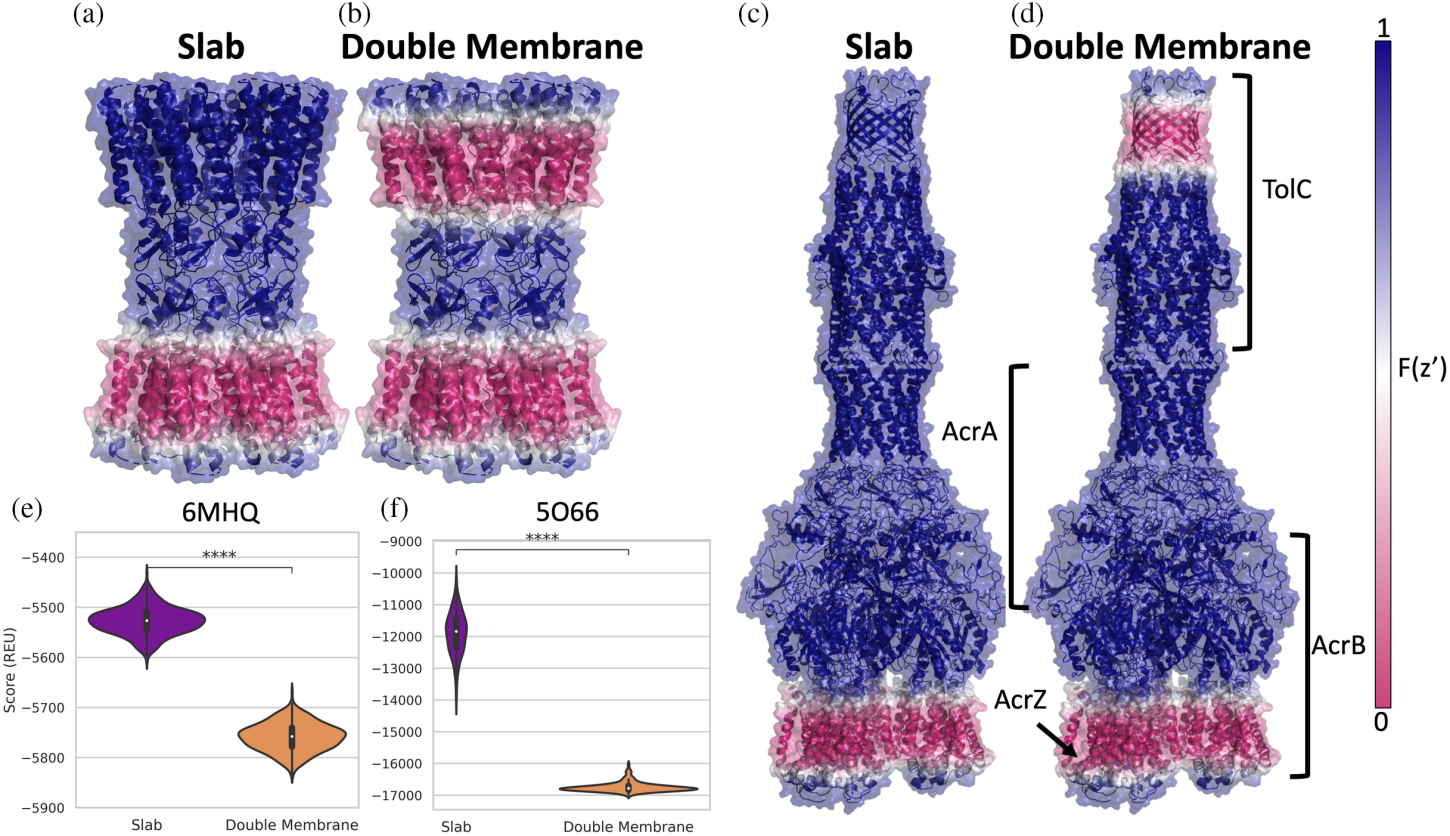
Refinement in double membrane. (a) An intercellular gap junction channel (PDB ID 6MHQ) with the slab transition function mapped onto the structure. A value of zero is shown in pink and indicates that residue is scored in a hydrophobic environment. A value of one is show in blue and indicates that residue is scored in an aqueous environment. (b) The same structure as in (a) with the double vesicle transition function mapped on to the structure. (c) AcrABZ‐TolC multidrug efflux pump (PDB ID 5O66) with the slab transition function mapped onto the structure. (d) The same structure as in (c) with the double vesicle transition function mapped onto the structure. (e) Score distributions for structural models created by running relax with the starting structure from PDB ID 6MHQ with franklin2019 in the slab (purple) and double vesicle (orange) geometries. (f) Score distributions for structural models created by running relax with the starting structure from PDB 5O66.

The first example we tested is a gap junction channel, where two transmembrane proteins in lipid bilayers of different cells are forming the gap junction. We used the connexin 46 gap junction channel (PDB ID 6MHQ) as a starting structure (Myers et al., 2018) and refined this structure in both the original slab membrane and in a double membrane. The value of the transition function was mapped onto the structure to visualize the membrane environment for each case (Figure 4a,b). The double membrane models all have lower scores than slab models (Figure 4e). This drop in score can be attributed to the *fa_water_to_bilayer* term which evaluates to zero when residues are sufficiently far from the membrane (Alford et al., 2020). Therefore, including the second membrane results in more residues with negative *fa_water_to_bilayer* values. The output models are similar for both geometries and the lowest scoring models overlap well with the starting structure (Figure S4a).

### 2.11 Implicit membranes for inner and outer membranes of gram‐negative bacteria

The second example we tested was the AcrABZ‐TolC efflux pump (PDB ID 5O66) (Wang et al., 2017). The transition function values for the slab (Figure 4c) and the double vesicle (Figure 4d) are mapped onto the structure to visualize the membrane for each case. In this complex, TolC is embedded in the outer membrane, while the AcrB and AcrZ subunits are embedded in the inner membrane (Figure 4d). The AcrA subunit is in the periplasm (Wang et al., 2017). Similar to the previous gap junction example, we see a drop in score with the double membrane simulations that can be partially attributed to more residues having a negative *fa_water_to_bilayer* scores since there is a second membrane (Figure 4f). Interestingly, the score distributions are much wider for models produced by slab geometry where there is a single membrane represented (Figures 4f and S4C). This results from sampling larger changes of the membrane position relative to the structure in the slab geometry compared to the double vesicle geometry, which anchors the complex better in the membrane. The larger range of scores is caused by the larger range of values in the *fa_water_to_bilayer* score term for the slab models than in the double membrane models. The output models generated by both geometries superimpose well with the starting structure and a similar tilt of TolC with respect to the rest of the complex is observed for both geometries (Figure S4B).

### 2.12 Incorporating experimental model membrane systems geometries in computation leads to more accurate models

For most structure determination methods, MPs must be extracted from the plasma membrane and reconstituted into a model membrane system. The composition and shape of the model membrane systems can impact the conformation of MPs. Having the ability to model MPs in geometries similar to the model membrane system in which the structure was determined in (or in which restraints were acquired) may provide additional insight into how model membrane systems introduce artifacts in MP structure and function. We use the ellipsoid model to capture the shape of model membrane systems including micelles and bicelles.

### 2.13 Protein–protein docking in experimentally relevant membrane geometry leads to better model discrimination

**FIGURE 5:**
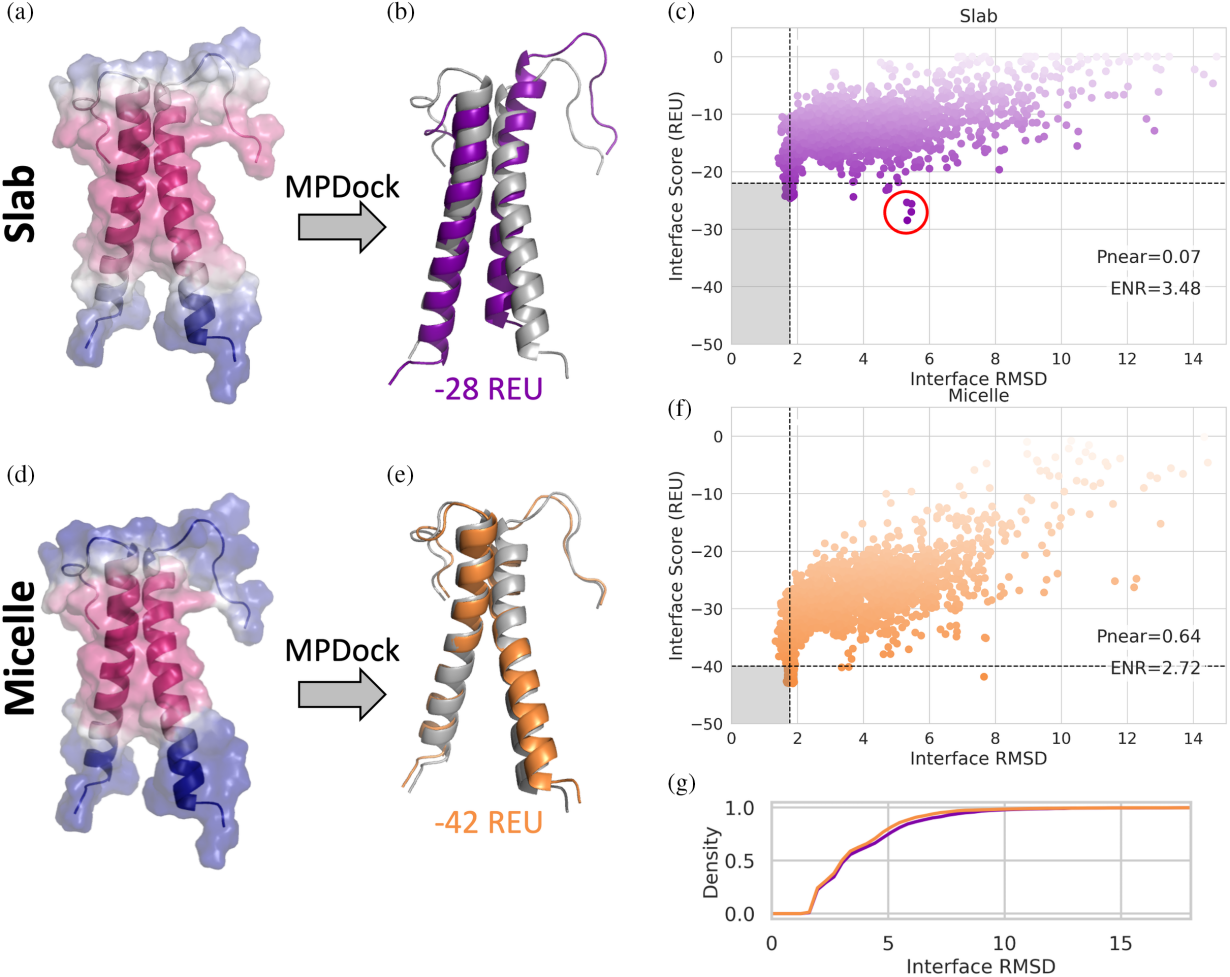
Docking with structure in membrane mimetics. (a) Glycophorin A dimer with slab transition function mapped onto the structure of the 1st conformation from the NMR ensemble from PDB ID 1AFO. (b) Lowest scoring structure after docking with slab geometry with an interface score of −28 REU. Starting structure, PDB 1AFO, shown in gray. (c) Interface RMSD and interface score from running MPdock protocol for PDB ID 1AFO in slab geometry, pnear value of 0.07 and enrichment value (ENR) of 3.48. Below the horizontal black dotted line are models that score in the lowest 10% according to InterfaceScore and to the left of the vertical black dotted line are models with the lowest 10% Interface RMSD values. The minimum based on interface score that identifies the incorrect interface is circled in red. (d) The same structure in (a) with the micelle geometry with inner radius 1 Å transition function mapped onto the structure. (e) Lowest scoring structure after docking with micelle geometry with an interface score of −42 REU. (f) Interface RMSD and interface score from running MPdock protocol for PDB ID 1AFO in bicelle geometry, pnear value of 0.64 and ENR value of 2.72. (g) Cumulative distribution of Interface RMSD of decoys produced by slab (purple) and Micelle (orange) geometries.

To demonstrate how modeling MPs in model membrane system geometries can be useful, we ran the protein–protein docking protocol, MPdock (Leman et al., 2015), on the glycophorin A homodimer, each with a single membrane spanning domain (Bormann et al., 1989). The structure of glycophorin A was determined by NMR in detergent micelles (MacKenzie et al., 1997). The MPdock protocol consists of two parts: a pre‐optimization step where the single chains are pulled apart and optimized, and the docking step. Each step was run using either the slab or micelle geometry. The transition function values for the slab and micelle geometries are mapped on the input structure (Figure 5a,d). The lowest scoring models for each geometry are superimposed onto the starting structure (PDB ID 1AFO) shown in gray (Figure 5b,e). The output models of the final step for each geometry are evaluated using the interface score versus the interface RMSD (Figure 5c,f), indicating that the models produced with the micelle geometry result in lower scores. The score function is also better able to discriminate lower RMSD models using the micelle geometry as indicated by the funnel metric (Pnear) value of 0.63 compared to 0.06 for the slab geometry. Pnear ranges from zero to one, where higher values indicate the metrics' (in this case the interface score) ability to favor conformations with lower RMSD value (Bhardwaj et al., 2016). While models produced by the slab and micelle geometries have the same RMSD distribution (Figure 5g), the incorrect interface would be identified as the best scoring model based on the interface score with the slab geometry, circled in red in Figure 5c. The correct interface is identified based on the interface score with the micelle geometry (Figure 5f). We see a similar effect with DockQ and fraction of native contacts recovered (Fnat) where more of the lowest scoring models produced with the micelle geometry also correspond to the models with higher DockQ and Fnat scores (Figure S5) (Basu & Wallner, 2016). The ability to use score to discriminate models more like the native docking pose is important because score is one of the main metrics used to rank and choose models for docking predictions you do not already know the answer to. According to the DockQ score classification used, the slab geometry produced 0 high quality, 637 medium quality, and 910 acceptable quality models out of 2500 total models. The micelle geometry produced 1 higher quality, 667 medium quality, and 961 acceptable quality out of 2500 total models.

### 2.14 Refinement of transmembrane helix in membrane geometry similar to experiment results in models more similar to experimental structure

**FIGURE 6:**
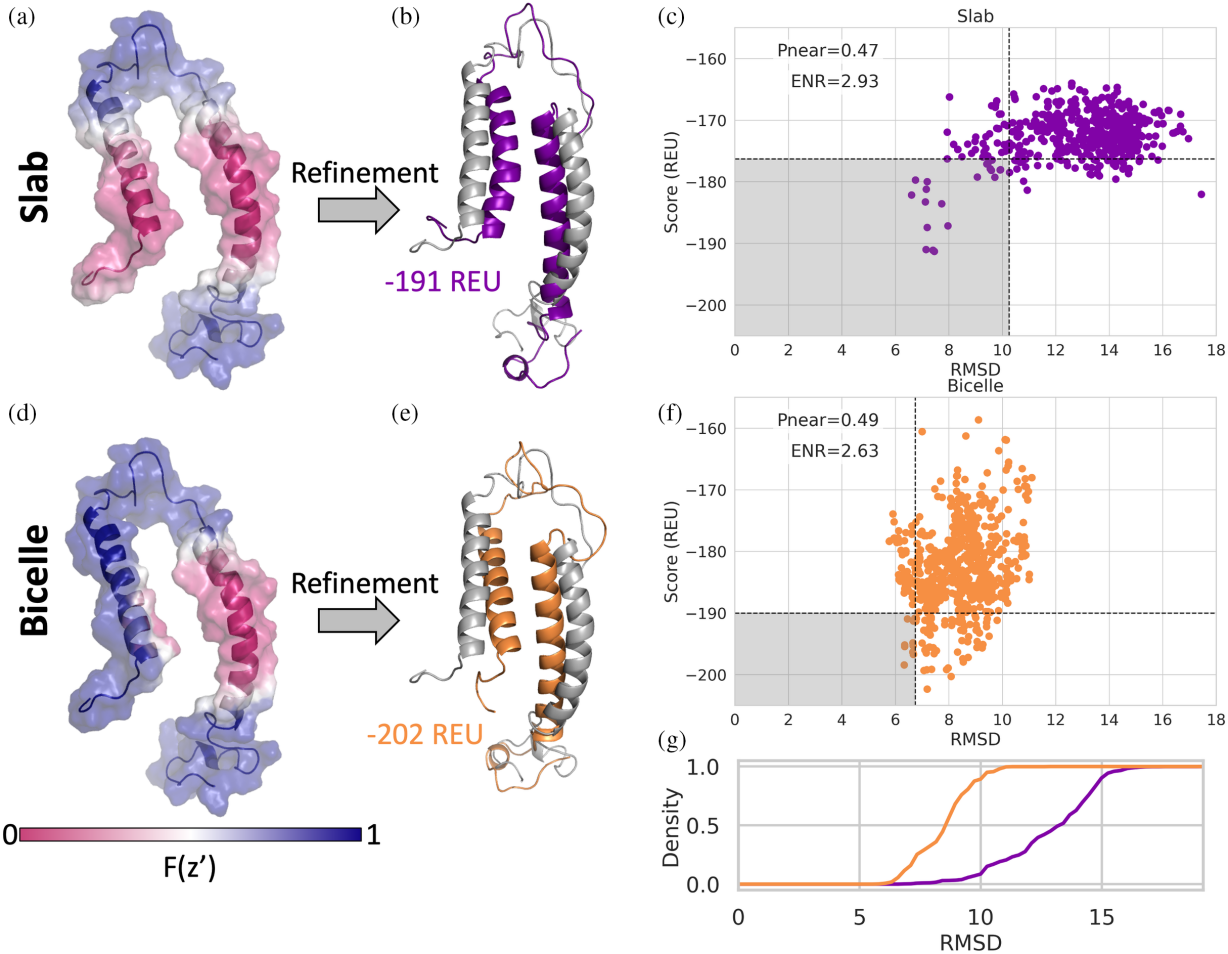
Refinement with structure in membrane mimetics. (a) KCNE3 with slab transition function mapped onto the structure of the 4th conformation in NMR ensemble PDB ID 2NDJ. (b) Lowest scoring structure after refinement with slab geometry with a score of −191 REU. Starting structure, PDB 2NDJ, shown in gray. (c) RMSD with respect to starting structure vs. score of models from refinement with slab geometry, Pnear value of 0.47 and enrichment value (ENR) of 2.93. Below the horizontal black dotted line are models that score in the lowest 10% and to the left of the vertical black dotted line are models with the lowest 10% RMSD values. (d) The same structure in (g) with the bicelle geometry with inner radius 2 Å transition function mapped onto the structure. (e) Lowest scoring structure after refinement with bicelle geometry with a score of −202 REU. (f) RMSD with respect to starting structure vs. score of models from refinement with bicelle geometry, pnear value of 0.49 and enrichment value of 2.63. (g) Cumulative distribution of RMSD of decoys produced by slab (purple) and bicelle (orange) geometries.

The structure of KCNE3 was determined with NMR in bicelles (Kroncke et al., 2016), showing a single span MP with an N‐terminal amphipathic helix. However, when scoring the models in the NMR ensemble with the traditional slab membrane model, some conformations such as the fourth conformation in the ensemble, are scored as if the N‐terminal amphipathic helix was dipping into the membrane (Figure 6a). We chose to work with the fourth conformation in the NMR ensemble to highlight how the ellipsoid geometry can be utilized to more accurately model the hydrophobic environment the protein is in. This scenario is more accurately modeled using the bicelle geometry, where the N‐terminal helix is now scored as if it is resting on the edge of the bicelle (Figure 6d). In fact, when this conformation is refined and scored using the slab and bicelle geometries, the bicelle geometry results in lower scoring models and lower RMSD values relative to the starting structure (Figure 6c,f,g). The relative position of the two helices with respect to each other changes for both geometries, bringing the helices closer together, which can be seen with the lowest scoring structures (Figure 6b,e), resulting in RMSD values ~7Å.

### 2.15 Representing geometry of model membrane systems allows separating artifacts from model membranes vs. structure determination method

**FIGURE 7:**
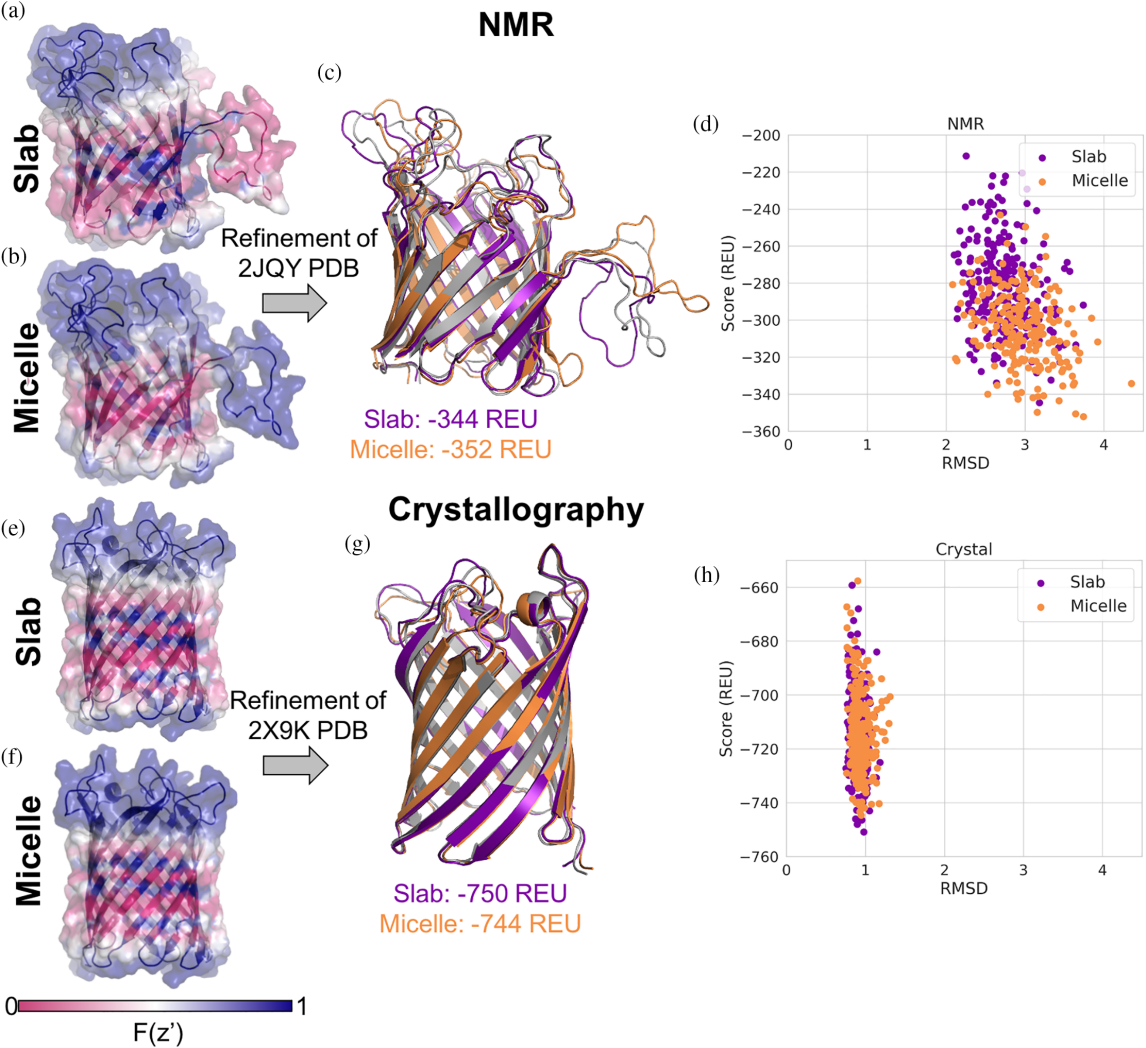
Refinement of OmpG NMR and crystal structures. (a) OmpG with slab transition function mapped onto the structure of the 1st conformation from the NMR ensemble from PDB ID 2JQY. (b) The same structure in (a) with the micelle geometry with inner radius 8 Å transition function mapped onto the structure. (c) Lowest scoring structure after refinement, model from slab geometry is purple with a score of −344 REU, model from micelle geometry is orange with a score of −352 REU, starting structure (PDB ID 2JQY) in gray. (d) RMSD with respect to starting structure vs. score of models from refinement, slab geometry in purple, micelle geometry in orange. (e) OmpG with slab transition function mapped onto the crystal structure PDB ID 2X9K. (f) The same structure in (e) with the micelle geometry with inner radius 8 Å transition function mapped onto the structure. (g) Lowest scoring structure after refinement, model from slab geometry is purple with a score of −750 REU, model from micelle geometry is orange with a score of −744 REU, starting structure (PDB ID 2X9K) in gray. (h) RMSD with respect to starting structure vs. score of models from refinement, slab geometry in purple, micelle geometry in orange.

While model membrane systems can impact the protein structure, even in similar systems different structure determination techniques can result in different conformations (Koehler Leman et al., 2018). For example, the structure of outer membrane protein G (OmpG) has been determined in micelles with both NMR (DPC micelles) (Liang & Tamm, 2007) and crystallography (OG micelles) (Korkmaz‐Ozkan et al., 2010). We ran refinement on the NMR model and crystal structure in both slab and micelle geometries. The aqueous environment of the pore is accounted for with both geometries, resulting in residues facing the pore having higher transition values (Figure 7a,b,e,f). The NMR model (PDB ID 2JQY) has a large loop that seemingly wraps around the micelle, but in the slab, geometry is treated like it is in the membrane (Figure 7a). In the micelle geometry with an inner radius of 8 Å this loop is scored like it is in an aqueous environment (Figure 7b). After refinement the models do not have large structural differences to the input structure (Figure 7c,d). However, the score distribution for the micelle geometry is shifted toward more negative scores as compared to the slab geometry (Figure 7d). Mapping the slab and micelle transition functions (micelle inner radius of 8 Å) onto the crystal structure (PDB ID 2X9K) does not result in large visual differences (Figure 7e,f). Refinement in both geometries sample similar structures with scores in the same range (Figure 7g,h).

## 3 DISCUSSION

We have implemented a general framework to adapt implicit membrane models to different geometries. Within this framework, there are currently four options available: slab, bicelle and micelle, vesicle, and two membranes. These geometries are compatible with existing score functions within the RosettaMP framework and allow users to accurately model MPs within specific environments based on their target.

The geometry framework was implemented to decouple the pore representation from the score functions, allowing older score functions within the MPFramework to be tested with the pore. We were able to analyze improvements of including the pore for both *franklin2019* and *mpframework2012* score functions. Sequence design metrics across all residues in the dataset only showed minimal differences when including the pore for either score function tested. However, inclusion of the pore only influences a handful of residues in each structure. When only considering pore‐facing residues, there is a significant difference for all three metrics when structures are designed with *franklin2019*, yet the difference for the *mpframework2012* score function is minor. *Franklin2019* has one membrane specific score term and this score term depends on the value of the transition function. *Mpframework2012* has three membrane score terms; only two depend on the transition function (fa_mpenv and fa_mpsolv). The other score term (fa_mpenv_smooth) is a knowledge‐based score term and does not depend on the transition function. While the change in the transition function from *franklin2019* without the pore (Figure S6A) to *franklin2019* with the pore (Figure S6C), and the change in the transition function from *mpframework2012* without the pore (Figure S6E) to *mpframework2012* with the pore (Figure S6G), are very similar, there seems to be a dampened effect on design results when including the pore with *mpframework2012* (Figures 2 and S2). Including the pore seems to increase the amount of non‐hydrophobic residues that are chosen during design for pore facing residues more for *franklin2019* (Figure S6B,D) than for *mpframework2012* (Figure S6F,H). Hydrophobic amino acids within the pore may still be scoring favorably due to the fa_mpenv_smooth score term that does not account for the pore. Franklin2019 may perform better with the pore since it was parameterized and tested with the pore implemented. On the other hand, *franklin2019* may overestimate the favorability of residues being buried in the membrane (Gulsevin & Meiler, 2021); therefore, scoring pore‐facing residues as being in an aqueous environment would help compensate for that.

We also compare design results from two additional score functions: *ref2015*_memb and *ref2015* (Figure S2). Ref2015_memb is a membrane specific score function based on experimental data (Elazar et al., 2016; Weinstein et al., 2019). It does not depend on a transition function, but instead is fit to data. Unfortunately, the pore model could not be adapted to this score function since there is not a corresponding transition function defined. Ref2015 is the current, standard soluble score function that franklin2019 and ref2015_memb are based on. As we would expect, there is no signification difference between design results of franklin2019 with the pore and ref2015 for aqueous and pore facing residue subsets for every metric except KL‐divergence of pore facing residues. Ref2015_memb and franklin2019 with the pore perform similar when comparing the design metrics tested (Figure S2). Ref2015_memb has higher sequence recovery for lipid facing residues (Figure S2A).

The vesicle geometry allows MPs to be modeled in a curved membrane, providing a more realistic representation of the MPs involved in membrane curvature. Membrane curvature may not be as important for small, compact MPs since the curvature would have to be high for the residues considered in the membrane to change from the slab to the vesicle representation. Large protein complexes are more likely to benefit from a curved membrane representation. When refining the Piezo 1 channel in both slab and vesicle, the slab model produces low‐quality output models where the protein essentially breaks apart. For the AlphaFold2 model of the TOM complex followed by Rosetta refinement, we see lower scoring and higher RMSD models for the simulations run with the curved membrane. Although the structures produced during refinement with the curved membrane have higher RMSDs compared to the slab membrane, the curve observed in the structures from both are similar to the starting structure (Figure 3j,n). The curved membrane results in more favorable scores since it is able to better match the membrane spanning region.

We can now model large complexes that span two membranes using a double vesicle geometry with a large radius. For these examples (gap junctions and ArcABZ‐TolC complex), there is a clear drop in score for systems when they are scored with the additional membrane. This is expected since the membrane domain of these complexes would be unstable if not embedded in the membrane. For the examples tested, the refinement protocol produces relatively tight ensembles with small differences in the RMSD of models.

MP structures are often determined in a model membrane system. Although the conformations scientists may be interested in are the ones in the native membranes, we typically only have access to structures in model membrane systems. Model membrane systems are often a surrogate for native membranes during structure determination experiments, but it is important to keep in mind that the model membrane affects the energy landscape of the MP. For example, the conformation of KCNE3 and OmpG chosen for refinement in Figures 6 and 7 are unlikely to exist in their respective native membrane environments. However, they are more likely to be a favored in model membrane systems such as micelles and bicelles used for membrane protein structure determination. With the introduced geometries, we can compare results between native and model membranes. Although the differences in scoring may be subtle, in some of the examples shown using the model membrane geometry resulted in lower scoring models. Being able to model different model membrane systems geometries might now allow us to separate effects from the model membrane system and the structure determination method, yet further studies are needed in this area.

In this work, we show how implicit membrane energy functions can be adopted to represent alternate membrane geometries instead of only a flat bilayer. Using appropriate geometries can improve both scoring and sampling. For most examples shown we observe lower scores when using the appropriate geometry. We observe narrower ranges in both RMSD and score of models being sampled when using the appropriate geometry, with the sampled conformations having lower RMSD, lower scores, or both indicating a more focused sampling of desired conformations.

## 4 CONCLUSION

Modeling and designing MPs continues to be a challenge in the protein structure field. However, many advances are being made to improve computational predictions for MPs. The complex and diverse environments MPs are in, contributes to this challenge. The framework introduced here allows modeling MPs in different geometries that cover experimentally used model membrane systems, curved membranes, and two membranes. The ability to model MPs in different model membrane systems opens the door for studies to separate the effect of model membrane systems and structure determination methods. The code design ensures the geometries, including the pore, are compatible with different energy functions, as well as different applications, such as refinement, docking, and design. These applications can be utilized with experimentally determined structures as well as structures generated by other prediction algorithms such as AlphaFold2. In many cases, this framework improves sampling and scoring of MPs.

## 5 METHODS

### 5.1 Implementation of implicit membrane geometries

The RosettaMP framework has score functions that include physics‐based score terms that depend on an atom's position relative to the membrane. This dependence on the membrane is achieved through a function that models the transition from the hydrophobic phase to the aqueous phase, referred to as the

(1) $$∆G_{\text{memb}}=\sum_{r=1}^{N_{\mathrm{res}}}\sum_{a=1}^{N_{\text{atom}\left(r\right)}}\left(1-f_{\mathrm{hyd}}\right)\left({∆G}_{w,l\left(a\right)}^{\text{atom}}\right)$$

transition function, adapted from IMM1 (Lazaridis, 2003). The transition function ranges from zero to one, where an

(2) $$f_{\mathrm{hyd}}=f_{\mathrm{thk}}+f_{\text{cavity}}-f_{\mathrm{thk}}*f_{\text{cavity}}$$

atom in the hydrophobic phase has a value of zero, and an atom far from the membrane has a value of one (Figure 1a). For example, in the fa_water_to_bilayer score term from *franklin2019* (Equation 1) the $f_{\mathrm{hyd}}$ is the transition function (Alford et al., 2020). The $f_{\mathrm{hyd}}$ function is a composition of two functions (Equation 2), one representing the transition along the membrane normal and the other represents an

(3) $$f_{\mathrm{hyd}}=f_{\mathrm{thk}}+h_{\text{bicelle}}-f_{\mathrm{thk}}*h_{\text{bicelle}}$$

$$\begin{array}{l} \text{where}\;h_{\text{bicelle}}=\frac{r^{\prime n}}{1+r^{\prime n}},r^{\prime }=\frac{r}{\text{radius}},\text{and}\\ r\;\text{is distance from center of ellipsoid} \end{array}$$

aqueous pore as described in He et al. (2013) and Alford et al. (2020) (Figure 1b). In Equation (2), $f_{\text{cavity}}$ describes the transition in the pore and $f_{\mathrm{thk}}$ describes the transition out of the bilayer. Two other membrane‐dependent score terms, fa_mpenv and fa_mpsolv, are based on the transition function used in IMM1 (Barth et al., 2007). Therefore, the first step in implementing new geometries was to adapt the existing transition functions to accommodate different geometries. The transition function can be adapted to an ellipsoid geometry using a composition of functions (Equation 3) (Figure 1c,d). In Equation (3), the radius is what we refer to as the outer radius of the bicelle (Figure 1d). The user sets the inner radius using the ‐mp:geo:bicelle_radius option. The inner radius corresponds to the radius of the planar section of the ellipsoid, before the curve of the edges (Figure 1d). For a micelle with no planar section, the inner_radius is 0. The outer radius is set as the inner radius + the thickness of the membrane. Commonly used micelle and bicelle sizes have been studied and values or equations that depend on the concentration of detergent and lipid to calculate the radius have been reported (Lipfert et al., 2007; Mineev et al., 2016). If the user does not set a bicelle radius, then one is estimated by calculating the largest distance found between two alpha carbons within 3 Å of the center of the membrane, dividing that distance in half and multiplying by three. This ensures that the protein is not modeled in a bicelle that is smaller than the protein itself, since the lipids or detergent molecules surround the protein so the size is dependent on the transmembrane domain of the protein (Sanders & Sonnichsen, 2006). Adaptation of the transition function to a sphere is described by Nepal, Leveritt III, and Lazaridis (Nepal et al., 2018) (Figure 1e). The origin continues to be at the center of the protein and in the middle of the membrane layers. The center of the sphere is at (0, 0, −radius) or (0, 0, +radius) depending on if the protein should be modeled with the membrane curved down (−radius) or the membrane curved up (+radius). The double bilayer geometry is a composition of two sphere equations (Figure 1f). The origin is at the center of the inner and outer spheres. We have the user set the radius of the inner sphere and the distance between the inner and outer sphere, specifically the distance from the outer edge of the inner membrane to the inner edge of the outer membrane. By having the distance parameter be defined at the distance between the edges of the membrane, this ensures that the membranes do not overlap, potentially creating unexpected behavior.

### 5.2 Applications to setup membrane geometries

We created two applications to help the user choose and visualize the appropriate geometry parameters. The mp_optimize_geometry_params application takes a PDB file, spanfile, and specified geometry as input and returns a text file with recommended parameters for the given geometry. A spanfile defines which regions of the protein are expected to span the membrane. The format of a spanfile and details on creating one can be found in the supplement. The application tries to best match the residues that are scored in a hydrophobic environment to the residues that are defined to span the membrane in the provided spanfile. The mp_transition_bfactor application helps the user visualize how their MP of interest is being scored in the implicit membrane by mapping the transition function value for each atom into the b factor column of a PDB file. The value in the b factor column can be visualized in biomolecule visualization software such as Pymol or Chimera. This application is useful for checking that the protein is positioned in the membrane and the membrane is shaped as expected before moving on to run other applications.

### 5.3 Choosing parameters for membrane geometries

For accurate results, the user will want to ensure they choose appropriate parameters for the membrane geometry depending on their system. The first step in choosing parameters for the membrane geometry is to decide on which geometry to model your protein in: slab, ellipsoid (micelle or bicelle), vesicle, or double membrane. Users should make this decision based on the system or the experimental setup for their use case. If the user is unsure if their MP of interest should be modeled in a curved membrane, the structure can be ran in PPM 3.0 to see if the protein is predicted to exist in a curved membrane (Lomize et al., 2022). Once deciding the geometry, the next step is creating a spanfile, which defines the residues that are expected to span the membrane. More details on a spanfile can be found in the supplement. Keep in mind, that the mp_optimize_geometry_params application is sensitive to which residues are defined in the spanfile, so it is important to have an accurate spanfile if you are planning on using that application. After creating the spanfile, the next step will depend on the chosen geometry. For a micelle or bicelle geometry, PPM 3.0 is not applicable, therefore, the user can use the mp_optimize_geometry params application to get an initial radius value. For a curved or double membrane, one can use either the PPM 3.0 or the mp_optmize_geometry_params application. The PPM 3.0 webserver was developed with the goal of estimating the expected radius of curvature and distance between two membranes, therefore, it is recommended to start with parameters from PPM 3.0 when possible. For a curved membrane, the radius of curvature provided by PPM 3.0 can be used as the vesicle radius. For a double membrane, PPM 3.0 provides a PDB file with HETATMS that represent the membranes. The distance between membranes can be calculated by determining the distance between the membranes using the position of the HETATMS (Figure S7). The mp_optimize_geometry_params application can also be used for getting initial, but keep in mind the parameters are recommended based on matching hydrophobic regions to the residues defined in the spanfile, not on physics‐based energy calculations. Once the initial parameters are decided, the user should visualize the membrane determined by these parameters by using the mp_transition_bfactor application and their preferred biomolecular visualization software. It may be necessary to finetune parameters through this visualization check. Additionally, for any application using the micelle or bicelle geometry, the greatest distance between any two points within 3 Å of the XY plane at the membrane center is calculated. If the inner radius is less than half that distance, then a warning is printed out that the protein may be larger than the bicelle/micelle. For most cases, the bicelle/micelle should be larger than protein at the membrane center; however, for some structures this is not true because of soluble domains wrapping around the model membrane systems such as for KCNE3.

### 5.4 Protein design with pore

The protein design analysis is based on the MP Sequence Recovery benchmark test implemented within the Rosetta test server framework (Alford et al., 2021; Koehler Leman et al., 2021). Since we were specifically looking at how inclusion of the pore affected the sequence recovery, we used a subset of the original dataset of only MPs that included a pore (Koehler Leman et al., 2017). The pore functionality was turned off using the ‐has_pore 0 flag. To note, by including this flag with either 0, 1, or empty, the pore is not calculated. We ran the fixed‐backbone design protocol three times with one designed sequence output for each case. The metrics reported in Figure 2 were calculated for each run and the average of the three independent runs is plotted. Residues were classified as pore‐facing if $f_{\text{cavity}}>0.1$ and $f_{\mathrm{thk}}<0.75$, from Equation (2).

### 5.5 Relax on piezo 1

We used PDB ID 6B3R for the starting structure of Piezo 1, downloaded from the OPM database. The preparation and orientation of the structure is described in detail in the supplemental protocol capture. After orientation a spanfile was generated using the mp_span_from_pdb Rosetta application. We used the mp_transition_bfactor application to visualize the implicit membrane on the structure. We ran the FastRelax protocol using the franklin2019 energy function with either the slab or vesicle geometry with a radius of 120 Å generating a total of 200 structures for each geometry.

### 5.6 AlphaFold2 and Rosetta relax on mitochondrial TOM complex

We used AlphaFold2 multimer (Richard et al., 2022) to generate initial decoys for the mitochondrial TOM complex. The chain sequences from PDB ID 6UCU were used as input (Tucker & Park, 2019), with five multimer predictions per model, making a total of 25 models. These models were used as input to a FastRelax protocol using the franklin2019 energy function with either the slab geometry or a vesicle geometry with a radius of 180 Å, based on reported radius of curvature from PPM 3.0 (Lomize et al., 2022). For each of the 25 AlphaFold2 predictions, 80 structures were generated by the FastRelax protocol for a total of 2000 structures for each geometry.

### 5.7 Relax on gap junction channel and AcrABZ‐TolC multidrug efflux pump in double membrane geometry

The structure for the gap junction channel was obtained by downloading PDB ID 6MHQ (Myers et al., 2018) from the OPM webserver (Lomize et al., 2006; Lomize et al., 2012). The spanfile created using mp_span_from_pdb application corresponds to the residues that span the inner membrane. The double_vesicle_distance was set at 40 and the inner vesicle radius was set to 1000 Å. We ran FastRelax protocol using *franklin2019* to produce a total of 450 structures in both the slab and double vesicle geometries.

The structure for the AcrABZ‐TolC multidrug efflux pump was obtained by downloading PDB ID 5O66 (Wang et al., 2017) from the OPM webserver (Lomize et al., 2012). The double_vesicle distance was set to 244 Å and the inner vesicle radius was 1000 Å. We ran FastRelax protocol using *franklin2019* to produce a total of 200 structures in both the slab and double vesicle geometries.

### 5.8 Docking glycophorin a in micelle geometry

To dock glycophorin A we followed the steps described in Alford, Samanta, and Gray (Alford et al., 2021) for protein–protein docking using the *franklin2019*. We chose to use the first conformation in the NMR ensemble for PDB ID 1AFO. We did both the prepacking and docking steps in the slab and bicelles geometries, so the geometry stayed consistent throughout the protocol. We created 50 models during the prepacking step and used the lowest scoring model as input into the docking step. We output 5000 models for the docking step. The bicelle inner radius was set at 1 Å.

### 5.9 Relax KCNE3 in bicelle geometry

The NMR conformational ensemble PDB ID 2NDJ (Kroncke et al., 2016) was downloaded from the PDB and from the OPM webserver. We chose to use the fourth conformation in the NMR ensemble since the amphipathic helix was positioned beside the transmembrane helix, where in the slab geometry it would be scored as if it was dipping into the membrane (Figure 6A). For both the slab and bicelles geometries 1000 output models were produced with the FastRelax protocol using *franklin2019*. The bicelles inner radius was set to 2 Å. The RMSD was calculated with respect to the starting structure, the 4th conformation from PDB ID 2NDJ.

## AUTHOR CONTRIBUTIONS

**Hope Woods:** Conceptualization; investigation; software; visualization; writing – original draft. **Julia Koehler Leman:** Conceptualization; project administration; writing – review and editing. **Jens Meiler:** Conceptualization; writing – review and editing; funding acquisition; supervision.

## Supporting information


**Data S1.** Supporting Information

**Data S2.** Supporting Information
