# Supplementary material for: Modeling membrane geometries implicitly in Rosetta
Source: Protein Sci. 2024 Feb 15;33(3):e4908. doi: 10.1002/pro.4908 (PMC10868433; doi:10.1002/pro.4908)
Supplement: Supplementary file 1 — Data S1. Supporting Information [file PRO-33-e4908-s002.docx]

# Modeling membrane geometries implicitly in Rosetta

Hope Woods^1,2^, Julia Koehler Leman^3^, and Jens Meiler^1,4,5^*

^1^ Center of Structural Biology, Vanderbilt University, Nashville, Tennessee, TN 37235, United States

^2^ Chemical and Physical Biology Program, Vanderbilt University, Nashville, Tennessee, TN 37235, United States

^3^ Center for Computational Biology, Flatiron Institute, New York, NY, USA

^4^ Department of Chemistry, Vanderbilt University, Nashville, Tennessee, TN 37235, United States

^5^ Institute for Drug Discovery, Leipzig University Medical School, Leipzig, 04103, Germany

* To whom correspondence should be addressed.

## Supplemental Materials

### Supplemental Figures


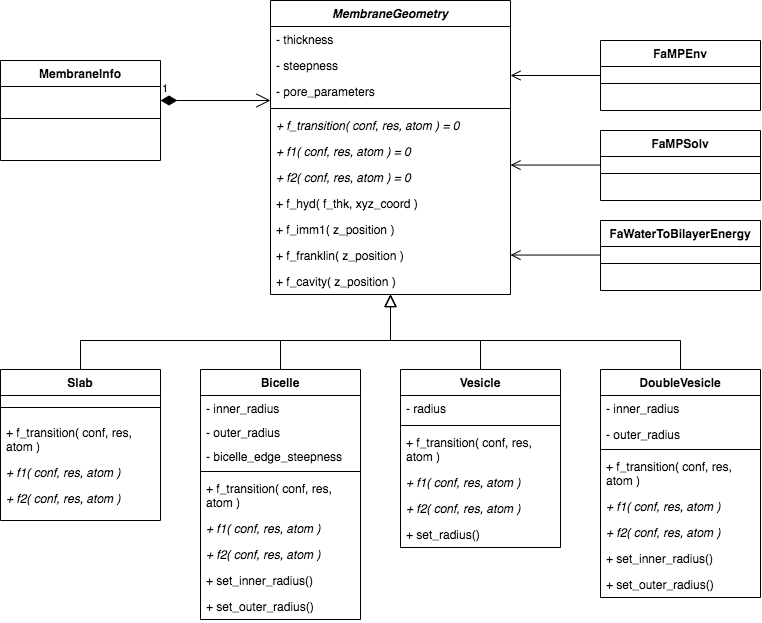


**Supplemental Figure S1** **UML diagram of MembraneGeometry Class.** Each block represents a different class. The top list members of that class and the bottom represents functions. Pure virtual functions, shown in italics, must be defined in all inherited classes. Not all members and functions for each class are shown. MembraneInfo contains an instance of MembraneGeometry. Slab, Bicelle, Vesicle, and DoubleVesicle are all inherited from MembraneGeometry. FaMPEnv, FaMPSolv, and FaWaterToBilayerEnergy are terms that associate with MembraneGeometry that all call f_transition to get the value of the transition function for each atom. F_imm1 is the transition function implemented with the mpframework2012 energy function. F_franklin is the transition function implemented with the franklin2019 energy function. Either of these can be combined with f_cavity to include the pore representation.


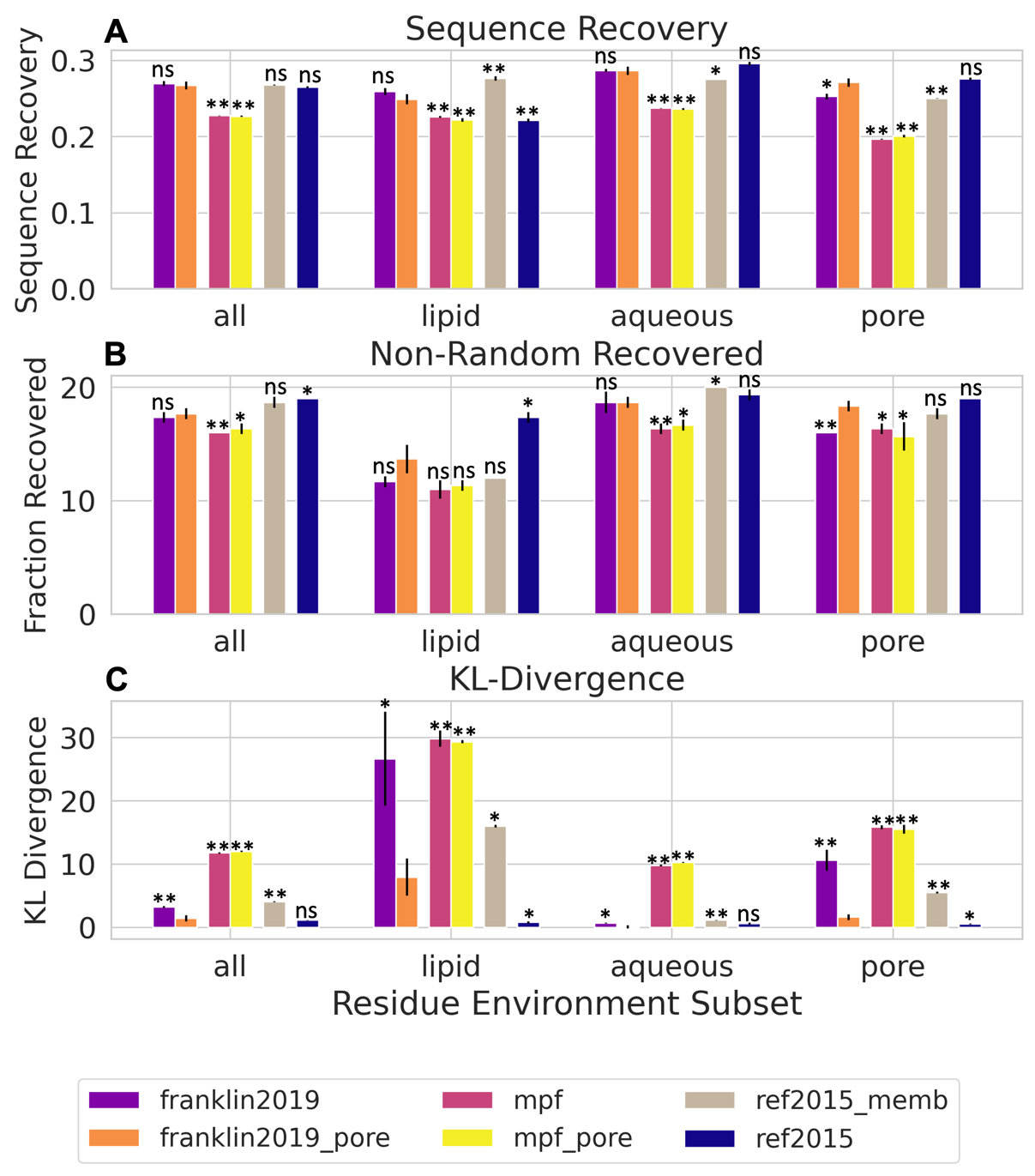


**Supplemental Figure S2: Sequence Design Comparison for Residue Subsets A)** Fraction of native residues recovered after design for all residues (left) lipid facing residues (middle left), aqueous facing residues (middle right), and pore facing residues (right). Higher values are expected for more accurate energy functions. Results using the *franklin2019* function are purple, *franklin2019* with the pore are orange, *mpframework2012* are pink, *mpframework2012* with the pore are yellow, *ref2015_memb* are beige, *ref2015* are dark blue. Significant difference from *franklin2019* with pore are donated with * where 0.01 < p <= 0.05 and ** where p <= 0.01.**B)** Average recovery rates for each individual amino acids relative to the background probability of randomly guessing the native amino acid (1/20). Higher values are expected for more accurate energy functions. **C)** Kullback-Leibler (KL) divergence measures how different distributions are of designed amino acids compared to native distributions. Lower values are expected for more accurate energy functions.


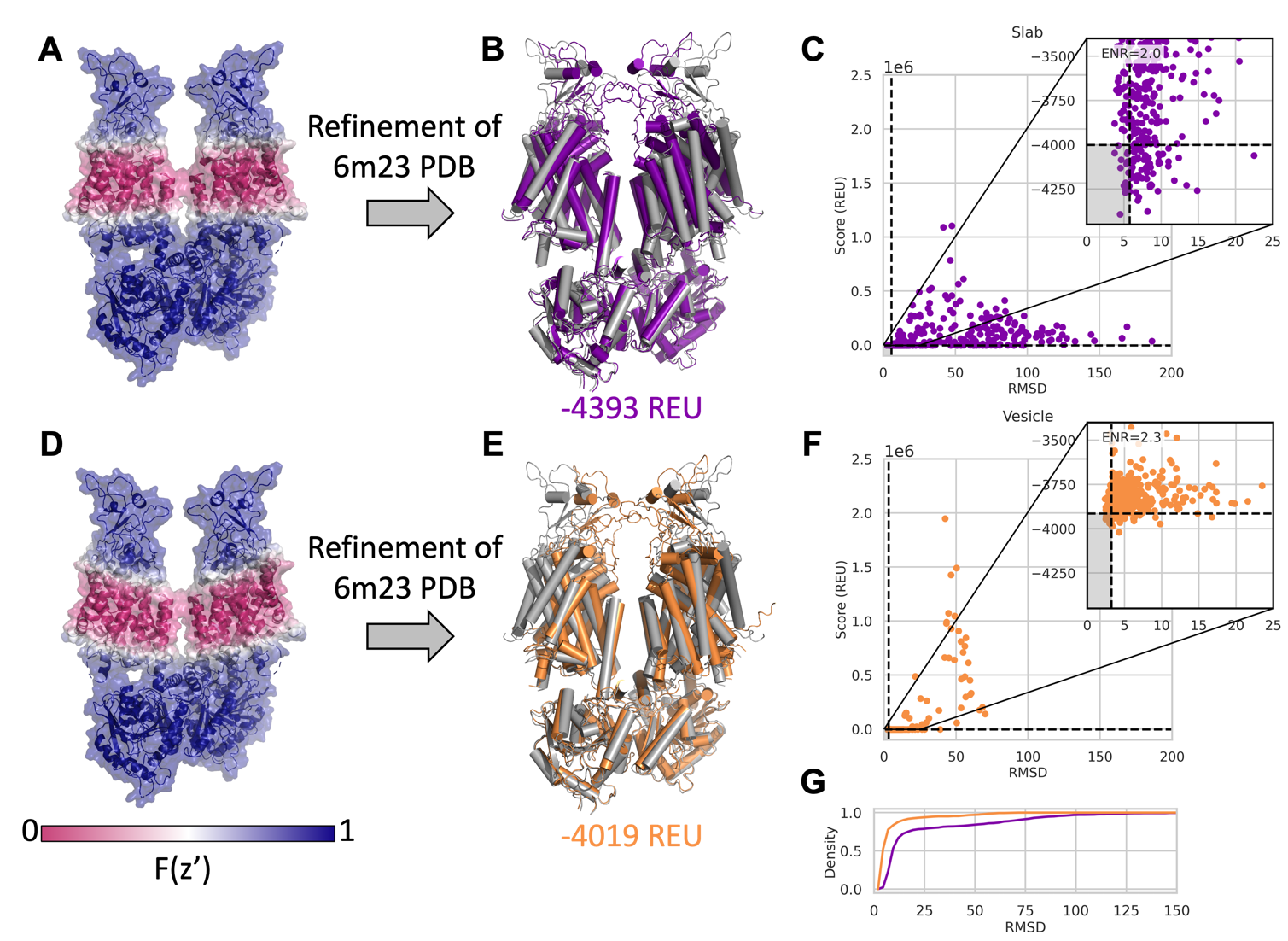


**Supplemental Figure S3 Refinement of KCC2 in Vesicle Geometry** A) Potassium chloride cotransporter KCC2 (PDB ID 6M23) with the slab transition function mapped onto the structure. B) Lowest scoring structure after refinement with slab geometry ran on PDB structure with a score of -4393 REU. Starting structure (PDB ID 6M23) shown in gray C) RMSD with respect to PDB ID 6M23 vs score of models from refinement with slab geometry, inset plot shows lowest scoring models. Below the horizontal black dotted line are models that score in the lowest 10% and to the left of the vertical black dotted line are models with the lowest 10% RMSD values. D) Same structure as A with Vesicle geometry transition function with a radius of 100 Angstroms. E) Lowest scoring structure after refinement with vesicle geometry ran on PDB structure with a score of -4019 REU F) RMSD with respect to PDB ID 6M23 vs score of models from refinement with vesicle geometry, inset plot shows lowest scoring models. G) Cumulative distribution of RMSD of decoys produced by slab (purple) and vesicle (orange)


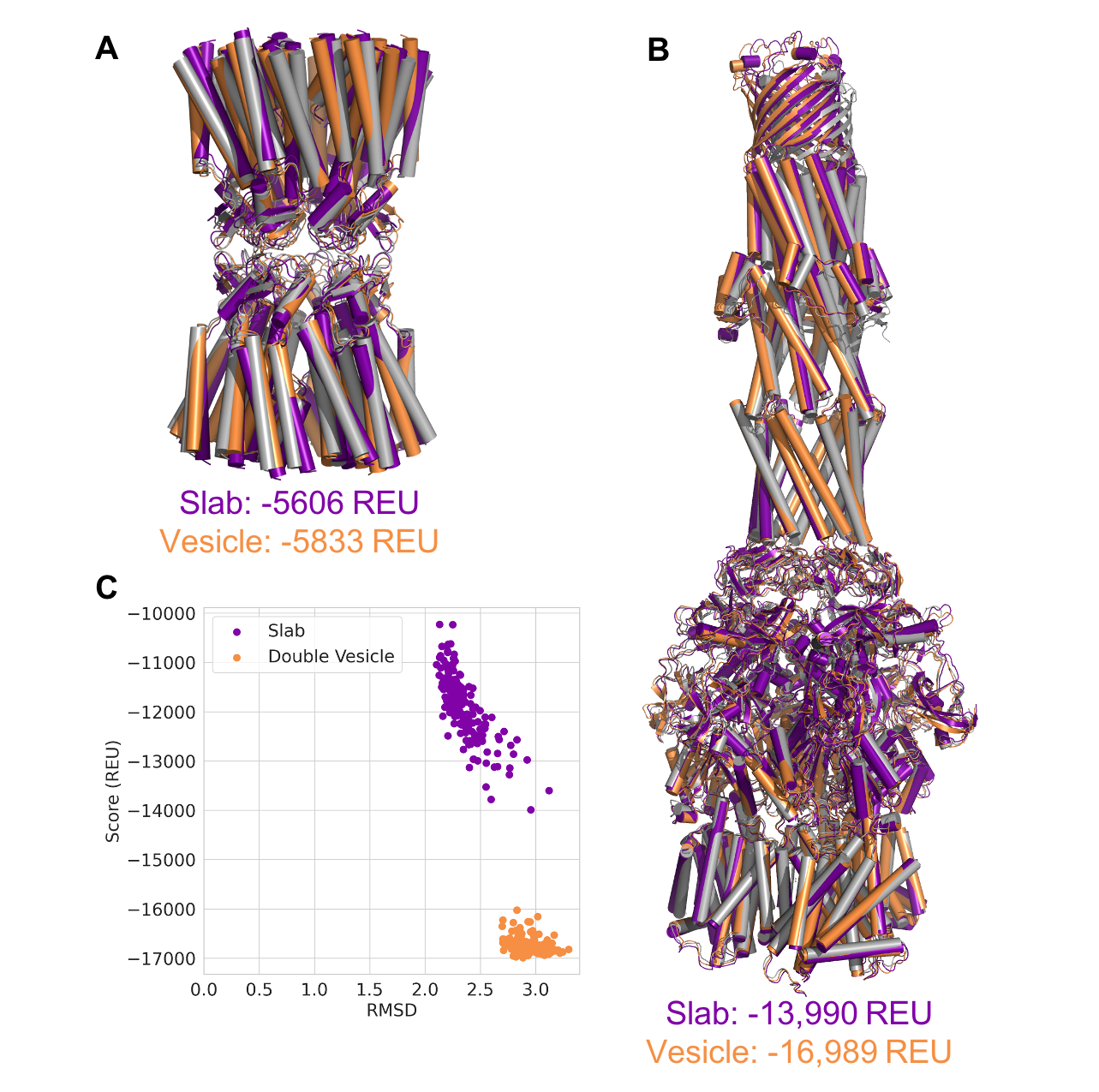


**Supplemental Figure S4 Double Vesicle Resulting** Models A) Lowest scoring structures from refinement of gap junction channel (PDB ID 6MHQ), slab in purple with a score of -5606 REU, double vesicle in orange with a score of -5833 REU, PDB ID 6MHQ in gray. B) Lowest scoring structures from refinement of efflux pump (PDB ID 5O66), slab in purple with a score of -13,990 REU, double vesicle in orange with a score of -16,989 REU, PDB ID 5O66 shown in gray. C) Efflux pump RMSD with respect to starting structure (PDB ID 5O66) vs score of models from refinement, slab geometry in purple, double vesicle geometry in orange.


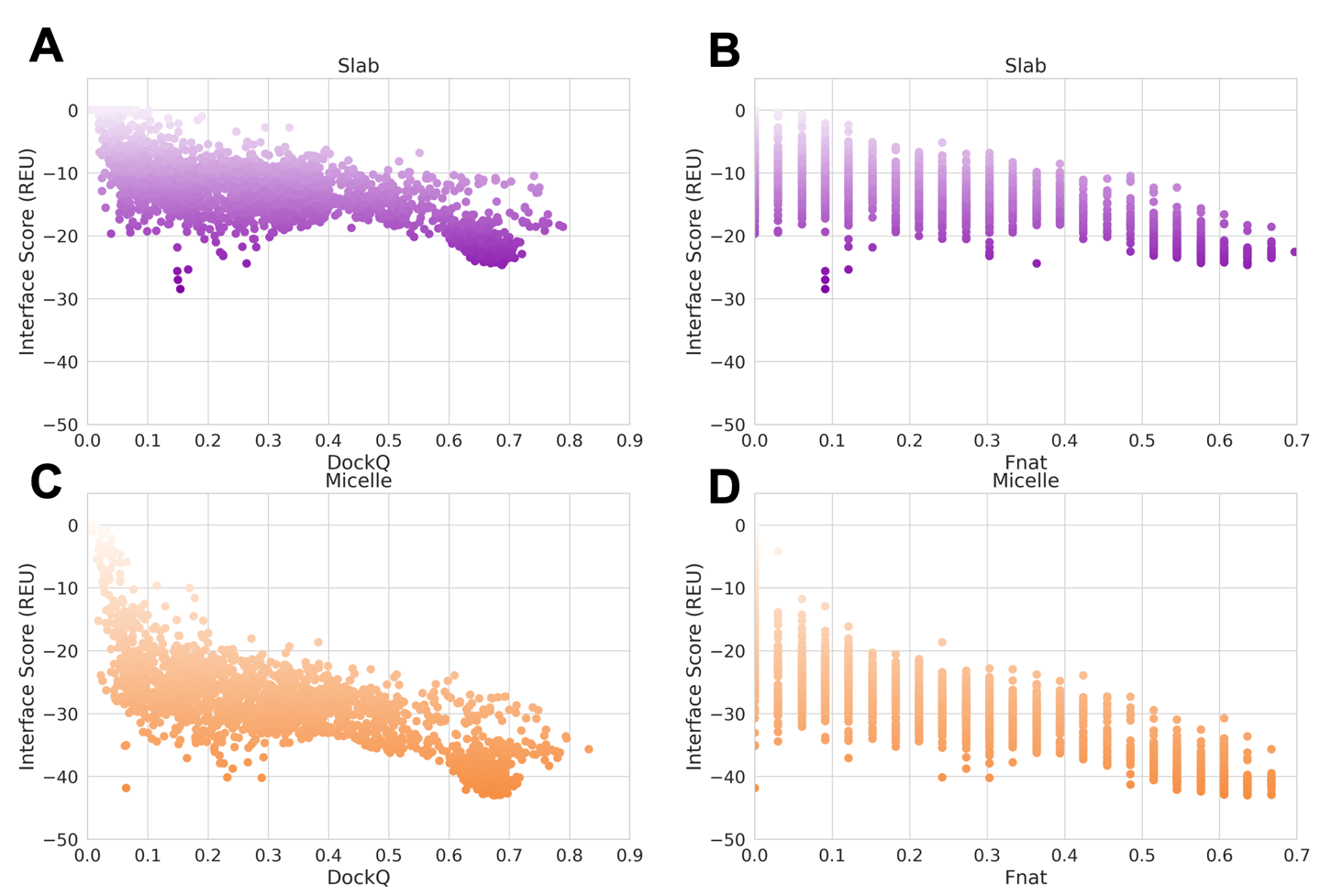


**Supplemental Figure S5: Docking Results for Glycophorin A A)** DockQ scores and Interface Score for docking results in slab geometry **B)** Fraction of native contacts recovered (Fnat) from docking results in slab geometry. **C)** DockQ scores and Interface Score for docking results in micelle geometry **D)** Fnat from docking results in micelle geometry


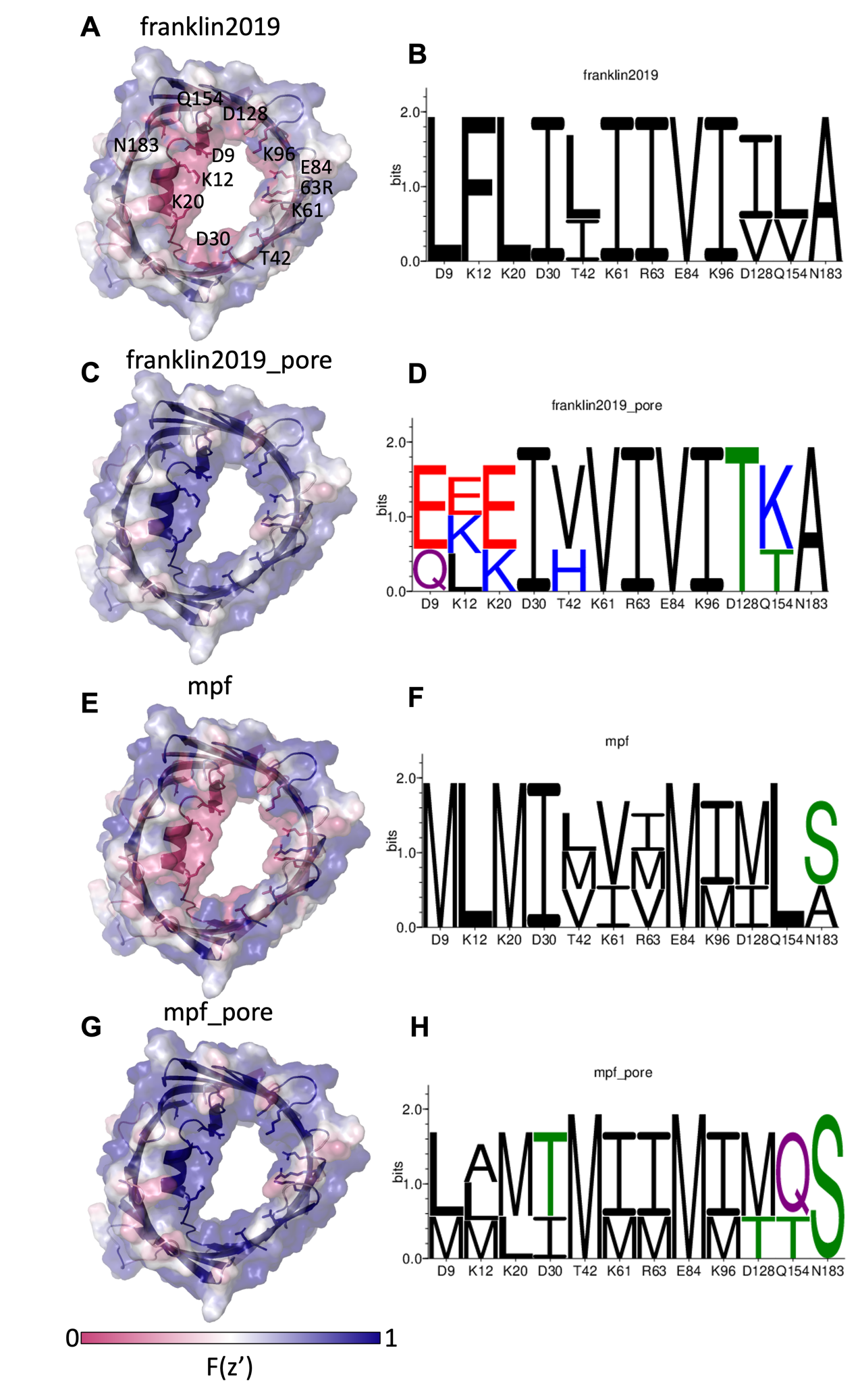


**Supplemental Figure S6: Transition Function and Design of VDAC** A) Transition function for *franklin2019* without the pore mapped onto voltage-dependent anion channel (VDAC) structure (PDB ID 3EMN), selected pore facing residues shown in sticks. B) Design results of selected pore facing residues (shown and labeled in A) using *franklin2019* without the pore. Hydrophobic amino acids colored black, polar amino acids colored green, neutral amino acids colored purple, basic amino acids colored blue, and acidic amino acids colored red. C) Transition function for *franklin2019* with the pore mapped onto VDAC structure. D) Design results using *franklin2019* with the pore. E) Transition function for *mpframework2012* without the pore mapped onto VDAC structure. F) Design results using *mpframework2012* without the pore. G) Transition function for *mpframework2012* with the pore mapped onto VDAC structure. H) Design results using *mpframework2012* with the pore.


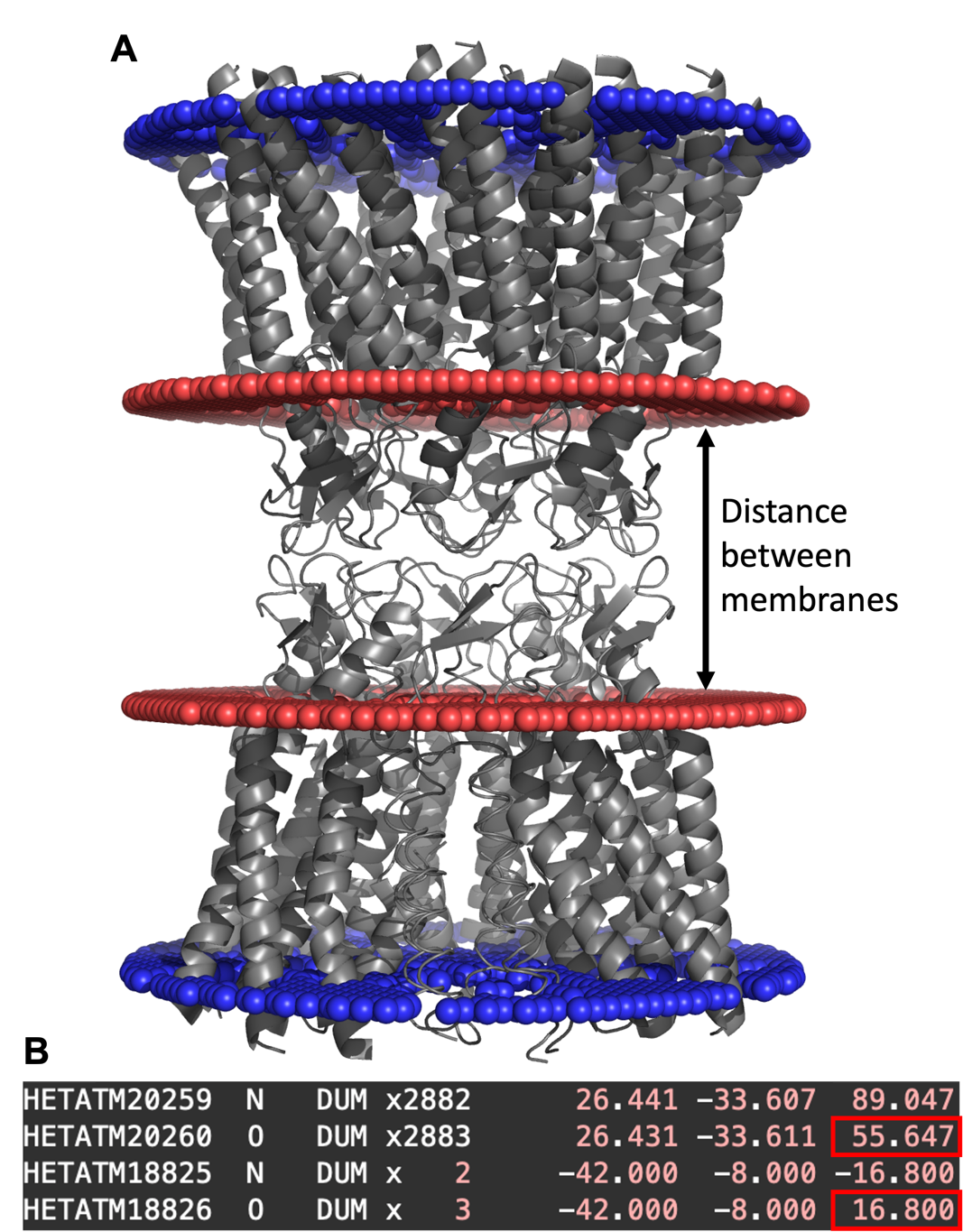


**Supplemental Figure S7:** **Predicted membranes from PPM 3.0** A) Predicted membranes from PPM 3.0 shown by red and blue spheres for outer and inner membranes respectively. B) Example of HETATM from the PDB file output by PPM 3.0. Red boxes are around the z-coordinates that can be subtracted to find the distance between the membranes.


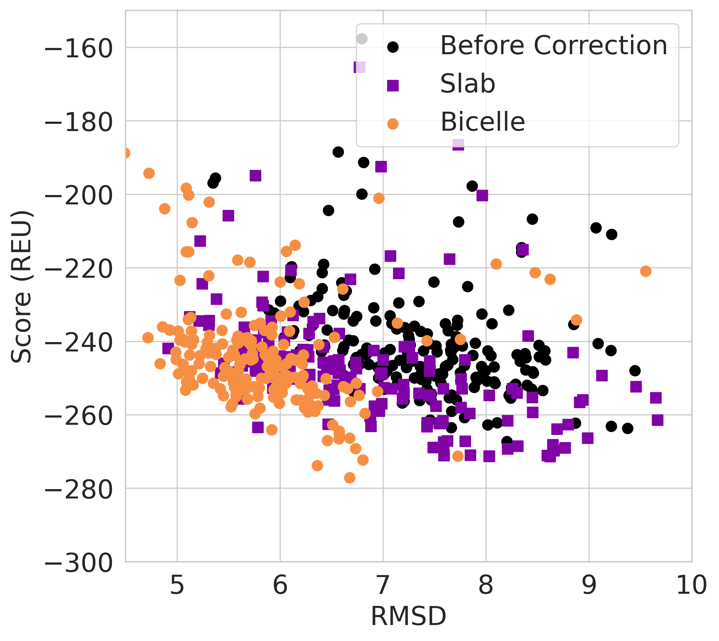


**Supplemental Figure S8 KCNE3 refinement with mpframework score function**. RMSD with respect to starting structure (PDB ID 2NDJ, 4th conformation) vs score of models from refinement done with the mpframework score function. Refinement with the score function before the derivative correction shown in black, after the derivative correction in the slab geometry in purple, and after the derivative correction in the bicelles geometry in orange.

## Supplemental Methods

### Code Design

To achieve our goals of flexible use across many applications and ease of further development, we implemented the new geometries as an interface class MembraneGeometry and each individual shape as an inherited class from MembraneGeometry (Figure S1). Score terms, such as FaMPEnv, FaMPSolv, and FaWaterToBilayerEnergy, that depend on an atom’s relative position to the membrane, the transition function value, get that information from MembraneGeometry. Previously, even for score terms that used the same transition function, that transition function was defined in the calculation for each score term. This creates a situation where one could be updated without updating all of them causing a mismatch in the implicit membrane being represented across score terms. This update ensures that the implicit membrane modeled is consistent across score terms.

The MembraneGeometry interface class houses members that are used across multiple geometries and virtual functions that must be defined in each inherited class. Thickness, steepness, and pore_parameters are variables that are needed across all geometries. Some geometry classes, such as Bicelle, have additional variables that are stored in the class itself, shown in Figure S1 in the top box of each geometry. There are three pure virtual functions contained in MembraneGeometry that must be defined in all inherited classes. These are shown in italics in the lower boxes for each class. The first pure virtual function is f_transition which returns the value of the transition function for an atom. The other virtual functions are $f1$ and $f2$. These are functions that allow for the calculation of the transition function derivative for gradient based minimization and are described in Abe et al (1) and this video: https://youtu.be/j07ibj-fT1A. For $f1$ and $f2$ one must first determine the point in space that the transition function value depends on the distance between the atom and that point, referred to as $r_{alpha}$. For example, if the membrane plane is at z=0 and the membrane normal is parallel to the z-axis, for the slab model $r_{alpha}$ for an atom at (x1, y1, z1) $r_{alpha}$ is (x1, y1, 0). If the derivative of the transition function with respect to an atom’s distance from $r_{alpha}$ is $\frac{dE}{dr}$, then $f1$ and $f2$ can be described as in equation S1 and S2. If the transition function depends on more than one distance, then $f1$ and $f2$ may need to contain multiple partial derivatives.

$f1=\frac{(r_{alpha} \times{atom}_{xyz})}{\left| r_{alpha}- {atom}_{xyz} \right|}*\frac{dE}{dr}$ (S1)

$f2=\frac{(r_{alpha}- {atom}_{xyz})}{\left| r_{alpha}- {atom}_{xyz} \right|}*\frac{dE}{dr}$ (S2)

To ensure proper implementation of each derivative, we implemented unit tests utilizing the Rosetta benchmarking framework (39) checking that the analytical and numerical derivatives are equal when calculated during minimization. This revealed some of the previous implementations of the derivatives needed to be updated. FaMPSolv energy term is a two-body term where the value depends both on an atom's relative distance to the membrane center and at atom’s distance to another atom (6; 3). The previous implementation only had the partial derivative with respect to the atom’s relative distance to the membrane center. We ran refinement on KCNE3 with the *mpframework2012* energy function that includes the FaMPSolv energy term and observed minor shifts in score and RMSD distributions with the slab geometry before and after the derivative correction (Figure S8). Refinement in the bicelle geometry impacts the distribution the most, like the impact using the franklin2019 energy function (Figure 6c and f).

### Membrane geometry parameter optimization application

The mp_optimize_geometry_params application returns the parameters for a given geometry that will create a membrane that best encompasses residues that are expected to be in the membrane based on the provided spanfile. The format for a spanfile can be found on the RosettaCommons website: https://www.rosettacommons.org/docs/latest/application_documentation/membrane_proteins/RosettaMP-GettingStarted-PreparingInputs. The residues that are expected to be in the membrane can either be determined by the user based on previous knowledge, experimental data, or predicted by various available software. TOPCONS is one example of a software that can be used for estimating which regions of the protein will span the membrane (61). Providing an accurate spanfile is crucial for this application to find good parameters. How the application determines the parameters is different for each geometry since each geometry has different parameters. For the curved membrane geometry, this application scans through radius values from -1000 and 1000. Negative radius values correspond to the membrane curving up and positive values the membrane curves down. The application returns the radius that has the highest rate of residues that are defined to be in the membrane according to the provided spanfile being in the membrane i.e., having a transition value greater than 0.5, what we call the true positive rate. If there are multiple radii that maximize the true positive rate, the application maximizes the true negative rate, and lastly the score. In case of the double membrane, the distance is first optimized in the same way with the inner_radius set at 1000 angstroms, and then the radius parameter is optimized. For the ellipsoidal membrane, the application scans through radius values starting at 0 and going up to twice the farthest distance between any two alpha carbons within 3 angstroms of the XY plane. This should ensure sampling of radius values sufficiently large to encompass the entire protein. For the ellipsoidal membrane, the product of the true negative and true positive rates is maximized, and the score is minimized if there are multiple radius values that maximize that product.

An example command line to use the optimization application for a bicelle geometry that will output a text file with the optimized parameters is provided below:

/Path/To/Rosetta/main/source/bin/mp_optimize_geometry_params. default.linuxgccrelease

-in:file:s PDB_FILE

-mp:setup:spanfiles SPAN_FILE

-mp:geometry bicelle

-in:membrane 1

### Sequence recovery with and without pore

The sequence recovery in Figure 2 is based on the benchmark test mp_f19_sequence_recovery on the Rosetta benchmark server [39, 51, 67]. When Rosetta detects a pore in an MP an output file describing the dimensions of the pore is created (file ends in .dat). We used proteins with a corresponding pore output file as input into the fixed backbone protein design. We used the PDB files and spanfiles in the test directory:

/Path/To/Rosetta/main/tests/scientific/data/mp_f19_sequence_recovery/

These PDB files were downloaded from the OPM database and spanfiles were created using mp_span_from_pdb application in Rosetta. Three of the pdb files were oligomers and the pore was being calculated at the interface of the subunits instead of where the pore was actually located: 1QD6, 3VY8, and 4KNF. These files were edited to only contain one subunit for an accurate pore representaiton. Fixed backbone protein design application was ran three times for each energy function with and without the pore on every protein in the subset with the command lines below. The comman lines below are examples with pdb 1EK9.

*Please note that including the flag -has_pore will result in overriding the pore calculation and no pore will be calculated regardless if you are setting this option to true/1 or false/0. This may be addressed in future versions.

*Also note when using the *mpframework2012* score function one should include the -mp:restore_lazaridis_imm_behavior true option to use the transition function implemented for the score terms in *mpframework2012*. Swapping the transition functions implemented for *mpframework2012* and *franklin2019* has not been properly benchmarked and should only be done for testing purposes or by experienced users.

Franklin2019 – there is no pore calcuated here:

/Path/To/Rosetta/main/source/bin/fixbb.default.linuxgccrelease

-in:file:s /Path/To/Rosetta/main/tests/scientific/data/mp_f19_sequence_recovery/1EK9/1EK9_tr_ignorechain.pdb

-mp:setup:spanfiles /Path/To/Rosetta/main/tests/scientific/data/mp_f19_sequence_recovery/1EK9/1EK9_tr_ignorechain.span

-mp:geometry slab

-score:weights franklin2019

-out:file:scorefile output/1EK9_franklin2019_nopore.sc

-nstruct 1

-in:membrane

-out:path output/

-out:prefix franklin2019_nopore_

-has_pore 0

> 1EK9_franklin2019_nopore.log

Franklin2019_pore – this includes the pore calculation:

/Path/To/Rosetta/main/source/bin/fixbb.default.linuxgccrelease

-in:file:s /Path/To/Rosetta/main/tests/scientific/data/mp_f19_sequence_recovery/1EK9/1EK9_tr_ignorechain.pdb

-mp:setup:spanfiles /Path/To/Rosetta/main/tests/scientific/data/mp_f19_sequence_recovery/1EK9/1EK9_tr_ignorechain.span

-mp:geometry slab

-score:weights franklin2019

-out:file:scorefile output/1EK9_franklin2019.sc

-nstruct 1

-in:membrane

-out:path output/

-out:prefix franklin2019_

> 1EK9_franklin2019.log

Mpf – there is no pore calculated here:

/Path/To/Rosetta/main/source/bin/fixbb.default.linuxgccrelease

-in:file:s /Path/To/Rosetta/main/tests/scientific/data/mp_f19_sequence_recovery/1EK9/1EK9_tr_ignorechain.pdb

-mp:setup:spanfiles /Path/To/Rosetta/main/tests/scientific/data/mp_f19_sequence_recovery/1EK9/1EK9_tr_ignorechain.span

-mp:geometry slab

-score:weights mpframework_smooth_fa_2012.wts

-mp:restore_lazaridis_imm_behavior true

-out:file:scorefile output/1EK9_mpf_nopore.sc

-nstruct 1

-in:membrane

-out:path output/

-out:prefix mpf_nopore_

-has_pore 0

> 1EK9_mpf_nopore.log

Mpf_pore – this includes the pore calculated:

/Path/To/Rosetta/main/source/bin/fixbb.default.linuxgccrelease

-in:file:s /Path/To/Rosetta/main/tests/scientific/data/mp_f19_sequence_recovery/1EK9/1EK9_tr_ignorechain.pdb

-mp:setup:spanfiles /Path/To/Rosetta/main/tests/scientific/data/mp_f19_sequence_recovery/1EK9/1EK9_tr_ignorechain.span

-mp:geometry slab

-score:weights mpframework_smooth_fa_2012.wts

-mp:restore_lazaridis_imm_behavior true

-out:file:scorefile output/1EK9_mpf.sc

-nstruct 1

-in:membrane

-out:path output/

-out:prefix mpf_

> 1EK9_mpf.log

The mp_seqrecov application was ran to calculate sequence recovery metrics for each run. This application included an edit that classified residues as pore facing if $f_{cavity}$ from equation 2 in the main text was greater than 0.1 and $f_{thk}$ from equation 2 was less than 0.75. Below is an example command line:

/Path/To/Rosetta/main/source/bin/mp_seqrecov.default.linuxgccrelease

-native_pdb_list native_pdbs.list

-redesign_pdb_list franklin2019_designed_pdbs.list

-seq_recov_filename franklin2019_fixbb_seqrecov.txt

-read_only_ATOM_entries

-in:ignore_unrecognized_res

> franklin2019_fixbb_seqrecov.log

The final metrics were calculated based on the benchmark test 2.analyze.py in the benchmark test folder, edited to inlcude the pore facing residues and average over the three independent runs:

/Path/To/Rosetta/main/tests/scientific/data/mp_f19_sequence_recovery/2.analyze.py

### Refinement in a curved membrane

To generate spanfiles for MPs in a slab geometry one can download the pdb file from the OPM database and run the mp_span_from_pdb application to create the spanfile for MPs in membrane coordinates [41, 45, 72]. For MPs that PPM 3.0 predicts to be optimally placed in a curved membrane, the OPM database has the center of the spherical membrane at the origin (0, 0, 0) instead of the center of the membrane at the origin. Because of this orientation no longer in RosettaMP membrane cooridnates, an additional step must be taken to orient the protien. If the radius as defined by PPM 3.0 on the OPM database is R then we use the mp_transform app to move the center of the membrane to (0, 0, R). For an MP predicted to be in a upward curved membrane R should be negative and a downward curved membrane R is positive.

To run refinment on Piezo 1 we first downloaded PDB 6B3R from the OPM database. This file was cleaned to remove any HETATM lines using the command line:

/Path/To/Rosetta/main/tools/protein_tools/scripts/clean_pdb.py 6b3r_opm.pdb ignorechain

We generated a dummy spanfile to be discarded later since the mp_transform application requires one to run using the mp_span_from_pdb application with the cleaned output pdb.

/Path/To/Rosetta/main/source/bin/mp_span_from_pdb.default.linuxgccrelease -in:file:s 6b3r_opm_ignorechain.pdb

We oriented the protein in the membrane using the mp_transform application. The OPM database reports the radius of curvature for 6b3r as 120 in a membrane curved down. Since the protein center was not at (0,0,0), but instead at (0,0,120), we used the following command to orient the protein where it is in the implicit membrane of Rosetta:

/Path/To/Rosetta/main/source/bin/mp_transform.default.linuxgccrelease -in:file:s 6b3r_opm_ignorechain.pdb -in:file:s 6b3r_opm_ignorechain.span --in:membrane -has_pore 0 -mp:setup:center 0 0 120

We use the following command to remove the virtual atoms output in the pdb file from the mp_transform application:

cat 6b3r_opm_ignorechain_0001.pdb | grep -v EMB | grep -v MEM > 6b3r_center120A.pdb

A new spanfile is created from the transformed pdb:

/Path/To/Rosetta/main/source/bin/mp_span_from_pdb.default.linuxgccrelease -in:file:s 6b3r_center120A.pdb

The implicit membrane was visualized by mapping the transition function value to the b factor column of a pdb file and the output structure was colored by b factor in pymol. The mp_transition_bfactor application will produce an output pdb with the same name as the input pdb, except it will add the mp_transition_ prefix to the file. Be sure to rename the file if you are producing multiple files with this application in the same directory or they will be overwritten each time.

Mapping the slab transition function with this command line:

/Path/To/Rosetta/main/source/bin/mp_transition_bfactor.default.linuxgccrelease

-in:file:s 6b3r_center120.pdb

-mp:setup:spanfiles 6b3r_center120.span

-mp:geometry slab

-score:weights franklin2019

-in:membrane

-has_pore 0

Mapping the vesicle transition function with this command line:

/Path/To/Rosetta/main/source/bin/mp_transition_bfactor.default.linuxgccrelease

-in:file:s 6b3r_center120.pdb

-mp:setup:spanfiles 6b3r_center120.span

-mp:geometry vesicle

-mp:geo:vesicle_radius -120

-score:weights franklin2019

-in:membrane

-has_pore 0

We ran relax with the franklin2019 score function with the slab geometry 200 times with the following command line:

/Path/To/Rosetta/main/source/bin/rosetta_scripts.default.linuxgccrelease @ options_frkln.txt

-nstruct 1

-out:prefix relax_slab_"$task"_

options_frkln.txt:

-parser:protocol ../../../membrane_relax.xml

-in:file:s ../6b3r_center120.pdb

-mp:setup:spanfiles ../6b3r_center120.span

-parser:script_vars scorefxn=franklin2019.wts

-mp:geometry slab

-has_pore 0

membrane_relax.xml:

*This is the same RosettaScript used for all refinement runs described in the mansuscript and protocols:

<ROSETTASCRIPTS>

<SCOREFXNS>

<ScoreFunction name="memb_hires" weights="%%scorefxn%%" />

</SCOREFXNS>

<MOVERS>

<AddMembraneMover name="add_memb"/>

<MembranePositionFromTopologyMover name="init_pos"/>

<FastRelax name="fast_relax" scorefxn="memb_hires" repeats="1"/>

</MOVERS>

<PROTOCOLS>

<Add mover="add_memb"/>

<Add mover="init_pos"/>

<Add mover="fast_relax"/>

</PROTOCOLS>

<OUTPUT scorefxn="memb_hires" />

</ROSETTASCRIPTS>

The RMSDs were calculated using the score_jd2 application:

/Path/To/Rosetta/main/source/bin/score_jd2.default.linuxgccrelease

-in:file:l pdb.list

-in:file:native ../6b3r_center120.pdb

-mp:setup:spanfiles ../6b3r_center120.span

-mp:geometry slab

-has_pore 0

-score:weights franklin2019

-no_nstruct_label 1

-out:prefix rmsd_starting_struct

-in:membrane 1

We ran relax with the franklin2019 score function with the vesicle geometry 200 times with the following command line:

/Path/To/Rosetta/main/source/bin/rosetta_scripts.default.linuxgccrelease @ options_frkln.txt -nstruct 1 -out:prefix relax_ves_"$task"_

options_frkln.txt:

-parser:protocol ../../../membrane_relax.xml

-in:file:s ../6b3r_center120.pdb

-mp:setup:spanfiles ../6b3r_center120.span

-parser:script_vars scorefxn=franklin2019.wts

-mp:geometry vesicle

-mp:geo:vesicle_radius -120

-has_pore 0

The RMSDs were calculated using the score_jd2 application:

/Path/To/Rosetta/main/source/bin/score_jd2.default.linuxgccrelease

-in:file:l pdb.list

-in:file:native ../6b3r_center120.pdb

-mp:setup:spanfiles ../6b3r_center120.span

-mp:geometry vesicle

-mp:geo:vesicle_radius -120

-has_pore 0

-score:weights franklin2019

-no_nstruct_label 1

-out:prefix rmsd_starting_struct

-in:membrane 1

The same steps were followed for refinement of KCC2, PDB 6M23 except a radius of -100 was used.

### Generating AlphaFold2 models and refinement in curved membrane

We used AlphaFold2 multimer to generate models of the mitochondrial TOM complex using the sequence of PDB 6UCU.

python $AF2_REPO/run_alphafold.py \

-fasta_paths=$FASTA \

--model_preset=multimer \

--num_multimer_predictions_per_model=5 \

--max_template_date=9999-12-31 \

--data_dir=$AF2_DATADIR \

--output_dir=$CALCDIR \

--use_gpu_relax \

--uniref90_database_path=$AF2_DATADIR/uniref90/uniref90.fasta \

--mgnify_database_path=$AF2_DATADIR/mgnify/mgy_clusters.fa \

--uniclust30_database_path=$AF2_DATADIR/uniclust30/uniclust30_2018_08/uniclust30_2018_08 \

--bfd_database_path=$AF2_DATADIR/bfd/bfd_metaclust_clu_complete_id30_c90_final_seq.sorted_opt \

--pdb_seqres_database_path=$AF2_DATADIR/pdb_seqres/pdb_seqres.txt \

--template_mmcif_dir=$AF2_DATADIR/pdb_mmcif/mmcif_files \

--obsolete_pdbs_path=$AF2_DATADIR/pdb_mmcif/obsolete.dat \

--uniprot_database_path /sb/apps/alphafold-data/uniprot/uniprot.fasta

Similar steps as above were taken to create a spanfile for the TOM complex using the PDB downloaded from the OPM database. We first cleaned it and then used the mp_transform application to move the membrane center to (0, 0, -180) since the predicted radius of curvature from PPM 3.0 was 180 angstroms and the membrane curved down. We aligned the output models from AlphaFold2 to the transformed pdb to place in membrane coordinates and used the aligned AlphaFold2 models as input into the refinement step. We used the -in:file:native flag to have the RMSD output to the score file for the refinement. The PDB file 6ucu was used as the native structure to calculate the RMSD to; however, the chains had to be renamed to match each AlphaFold2 output model. Chains were renamed manually through visual inspection in pymol.

An example command line for refinement of the AlphaFold2 models in the slab geometry is below, a total of 2000 models were generated.

/Path/To/Rosetta/main/source/bin/rosetta_scripts.default.linuxgccrelease

-in:file:s ../ranked_0_aligned.pdb

-mp:setup:spanfiles ../../6ucuout_ignorechain.span

-has_pore 0

-mp:geometry slab

-parser:protocol ../membrane_relax.xml

-parser:script_vars scorefxn=franklin2019.wts

-in:file:native NATIVE

-out:file:silent relax0_slab_0.out

-out:file:scorefile relax0_slab_0.sc

An example command line for refinement of the AlphaFold2 models in the vesicle geometry is below, a total of 2000 models were generated.

/Path/To/Rosetta/main/source/bin/rosetta_scripts.default.linuxgccrelease

-in:file:s ../ranked_0_aligned.pdb

-mp:setup:spanfiles ../../6ucuout_ignorechain.span

-has_pore 0

-mp:geometry vesicle

-mp:geo:vesicle_radius 180

-parser:protocol ../membrane_relax.xml

-parser:script_vars scorefxn=franklin2019.wts

-in:file:native NATIVE

-out:file:silent relax0_ves_0.out

-out:file:scorefile relax0_ves_0.sc

### Refinement of MP complexes in two membranes

For refinement of the gap junction channel the PDB 6MHQ was downloaded from the OPM database. The PDB was cleaned:

/Path/To/Rosetta/main/tools/protein_tools/scripts/clean_pdb.py 6mhq.pdb ignorechain

A spanfile was generated using the mp_span_from_pdb application:

/Path/To/Rosetta/main/source/bin/mp_span_from_pdb.default.linuxgccrelease -in:file:s 6mhq_ignorechain.pdb

This spanfile corresponds to regions in the lower membrane of the complex. The distance between the two membranes was estimated based on the distance between the z-coordinates of the HETATMS representing the two outer-membrane regions that are in the PDB file downloaded from OPM. The outer membrane is determined by setting the option -mp:geo:double_vesicle_distance to the distance from the outer edge of the inner membrane to the inner edge of the outer membrane. For refinement of 6MHQ, the double_vesicle_distance was set at 40 and the inner vesicle radius was set to 1000 Å, making the membrane practically flat.

The implicit membrane was visualized with the mp_transition_bfactor application and pymol as described above.

Mapping the slab transition function with this command line:

/Path/To/Rosetta/main/source/bin/mp_transition_bfactor.default.linuxgccrelease

-in:file:s 6mhq_ignorechain.pdb

-mp:setup:spanfiles 6mhq_ignorechain.span

-mp:geometry slab

-has_pore 0

Mapping the double vesicle transition function with this command line:

/Path/To/Rosetta/main/source/bin/mp_transition_bfactor.default.linuxgccrelease

-in:file:s 6mhq_ignorechain.pdb

-mp:setup:spanfiles 6mhq_ignorechain.span

-mp:geometry double_vesicle

-mp:geo:vesicle_radius 1000

-mp:geo:double_vesicle_distance 40

-has_pore 0

Below is an example command line for refinement. 450 models for each geometry were ouptut.

/Path/To/Rosetta/main/source/bin/rosetta_scripts.default.linuxgccrelease @ options_frkln.txt

options_frkln.txt for slab geometry:

-parser:protocol ../membrane_relax.xml

-in:file:s ../../6mhq_ignorechain.pdb

-mp:setup:spanfiles ../../6mhq_ignorechain.span

-parser:script_vars scorefxn=franklin2019.wts

-mp:geometry slab

-has_pore 0

options_frkln.txt for double vesicle geometry:

-parser:protocol ../membrane_relax.xml

-in:file:s ../../6mhq_ignorechain.pdb

-mp:setup:spanfiles ../../6mhq_ignorechain.span

-parser:script_vars scorefxn=franklin2019.wts

-mp:geometry double_vesicle

-mp:geo:vesicle_radius 1000

-mp:geo:double_vesicle_distance 40

-has_pore 0

The score_jd2 app using 6mhq_ignorechain.pdb as the native structure was used to calculate RMSD.

Refinement for the AcrABZ-TolC multidrug efflux pump was ran with the same steps using PDB 5O66 from the OPM database. The distance between membranes was estimated from the outer leaflet HETATM z-coordinate of the lower membrane and the inner leaflet HETATM z-coordinate of the upper membrane. The mp:geo:double_vesicle_distance used for refinement of the efflux pump in the double vesicle geometry was 244 angstoms.

### Protein-protein docking in micelle

For glycophorin A the PDB file was downloaded both from the RCSB and the OPM database because the OPM database only has one conformation from the NMR ensemble. The PDB file from RCSB was aligned to the PDB from OPM database to orient the structure into membrane coordinates. The aligned structure from the PDB was then cleaned with clean_pdb.py and a spanfile generated with mp_span_from_pdb application as described above. The first conformation in the NMR ensemble was used as starting structures for the MPDock protocol [42].The entire protocol was ran with both the slab and micelle geometry.

The first step in the MPDock protocol is prepacking. The command line for the prepacking step with the slab geometry used, with 50 structures output:

/Path/To/Rosetta/main/source/bin/docking_prepack_protocol.default.linuxgccrelease

-in:file:s ../1afo_aligned_ignorechain.pdb

-mp:setup:spanfiles ../1afo_aligned_ignorechain.span

-out:file:scorefile prepack_output/score_ppk_1afo_slab_fr.sc

-out:path prepack_output

-nstruct 50

-mp:geometry slab

-docking:partners A_B

-score:weights franklin2019

-mp:lipids:composition DLPC

-packing:pack_missing_sidechains 0

The lowest scoring structure from the prepacking step was used as the input structure for the mp_dock application. The command line for mp_dock with the slab geometry used, with a total of 5000 structures output:

/Path/To/Rosetta/main/source/bin/mp_dock.default.linuxgccrelease

-in:file:s prepack_output/1afo_prepack_slab_topscore.pdb

-in:file:native ../1afo_aligned_ignorechain.pdb

-mp:setup:spanfiles ../1afo_aligned_ignorechain.span

-mp:geometry slab

-score:weights franklin2019

-mp:lipids:composition DLPC

-out:file:silent 1afo_mpdock_slab.out

-out:file:scorefile 1afo_mpdock_slab.sc

-docking:partners A_B

-dock_pert 3 8

The radius used for the micelle geometry was estimated by visually inspecting the transition function with different radii mapped onto the structure. An inner radius of 1 angstrom was chosen since that scores the n-terminus as if it was in an aqueous environment instead of the membrane (Figure 5d). The command line for the prepacking step with the micelle geometry:

/Path/To/Rosetta/main/source/bin/docking_prepack_protocol.default.linuxgccrelease

-in:file:s ../1afo_aligned_ignorechain.pdb

-mp:setup:spanfiles ../1afo_aligned_ignorechain.span

-out:file:scorefile prepack_output/score_ppk_1afo_bicelle1_fr.sc

-out:path prepack_output

-nstruct 50

-mp:geometry bicelle

-mp:geo:bicelle_radius 1

-docking:partners A_B

-score:weights franklin2019

-mp:lipids:composition DLPC

-packing:pack_missing_sidechains 0

Again, the lowest scoring structure from the prepacking step was used as the input structure for the mp_dock application. The command line for mp_dock with the micelle geometry:

/Path/To/Rosetta/main/source/bin/mp_dock.default.linuxgccrelease

-in:file:s prepack_output/1afo_prepack_micelle_topscore.pdb

-in:file:native ../1afo_aligned_ignorechain.pdb

-mp:setup:spanfiles ../1afo_aligned_ignorechain.span

-mp:geometry bicelle

-mp:geo:bicelle_radius 1

-score:weights franklin2019

-mp:lipids:composition DLPC

-out:file:silent 1afo_mpdock_bicelle1.out

-out:file:scorefile 1afo_mpdock_bicelle1.sc

-nstruct 100

-docking:partners A_B

-dock_pert 3 8

### Refinement in micelle

We downloaded PDB files for KCNE3, PDB ID 2NDJ, from both the RCSB and the OPM database. Similar to glycophorin A, the structure from the RCSB was aligned to the OPM structure. We used the 4^th^ conformation from the NMR ensemble as a starting structure to highlight the importance of using the bicelle geometry. An inner radius of 2 angstroms was determined based on visual inspection so that the n-terminal helix that is not a transmembrane helix is scored like it is in an aqueous environment (Figure 6a). After the aligned pdb file was cleaned and a spanfile was created, refinement in the slab and bicelle geometry was ran.

Command line for KCNE3 refinement in slab geometry, with a total of 1000 output models:

/Path/To/Rosetta/main/source/bin/rosetta_scripts.default.linuxgccrelease

-parser:protocol ../membrane_relax.xml

-in:file:s ../prep/4_2ndj_mp.pdb

-in:file:native ../prep/4_2ndj_mp.pdb

-mp:setup:spanfiles ../prep/2ndj_opm_A.span

-parser:script_vars scorefxn=franklin2019.wts

-mp:geometry slab

-out:file:silent 2ndj_4_relax_slab_frkln.out

-out:file:scorefile 2ndj_4_relax_slab_frkln.sc

Command line for KCNE3 refinement in bicelle geometry, with a total of 1000 output models:

/Path/To/Rosetta/main/source/bin/rosetta_scripts.default.linuxgccrelease

-parser:protocol ../membrane_relax.xml

-in:file:s ../prep/4_2ndj_mp.pdb

-in:file:native ../prep/4_2ndj_mp.pdb

-mp:setup:spanfiles ../prep/2ndj_opm_A.span

-parser:script_vars scorefxn=franklin2019.wts

-mp:geometry bicelle

--mp:geo:bicelle_radius 2

-out:file:silent 2ndj_4_relax_bicelle_frkln.out

-out:file:scorefile 2ndj_4_relax_bicelle_frkln.sc

The same steps were taken to run refinement for OmpG.
